# Wadsley–Roth Crystallographic Shear Structure Niobium‐Based Oxides: Promising Anode Materials for High‐Safety Lithium‐Ion Batteries

**DOI:** 10.1002/advs.202004855

**Published:** 2021-03-15

**Authors:** Yang Yang, Jinbao Zhao

**Affiliations:** ^1^ School of Chemical Engineering and Light Industry Guangdong University of Technology Guangzhou 510006 P. R. China; ^2^ State Key Lab of Physical Chemistry of Solid Surfaces State‐Province Joint Engineering Laboratory of Power Source Technology for New Energy Vehicle College of Chemistry and Chemical Engineering Xiamen University Xiamen 361005 P. R. China

**Keywords:** anode materials, battery safety, high power density, lithium‐ion battery, Wadsley–Roth phase

## Abstract

Wadsley–Roth crystallographic shear structure niobium‐based oxides are of great interest in fast Li^+^ storage due to their unique 3D open tunnel structures that offer facile Li^+^ diffusion paths. Their moderate lithiation potential and reversible redox couples hold the great promise in the development of next‐generation lithium‐ion batteries (LIBs) that are characterized by high power density, long lifespan, and high safety. Despite these outstanding merits, there is still extensive advancement space for further enhancing their electrochemical kinetics. And the industrial feasibility of Wadsley–Roth crystallographic shear structure niobium‐based oxides as anode materials for LIBs requires more systematic research. In this review, recent progress in this field is summarized with the aim of realizing the practical applications of Wadsley–Roth phase anode materials in commercial LIBs. The review focuses on research toward the crystalline structure analyses, electrochemical reaction mechanisms, modification strategies, and full cell performance. In addition to highlighting the current research advances, the outlook and perspective on Wadsley–Roth anode materials is also concisely provided.

## Graphical abstract

Recent progress of developing Wadsley‐Roth phase anode materials are summarized. The review focus on research toward the crystalline structure analyses, electrochemical reaction mechanisms, modification strategies, and full cell performance. With the aim of realizing the practical applications of Wadsley‐Roth phase anode materials in commercial LIBs, the outlook and perspective is also concisely provided.

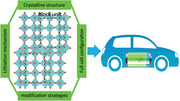

## 1 Introduction

The Nobel Prize in Chemistry 2019 was jointly awarded to Akira Yoshino, John B. Goodenough, and M. Stanley Whittingham for the development of lithium‐ion batteries (LIBs).^[^ ^1^ ^]^ Since its successful commercialization in 1991 by the SONY Corporation, this lightweight, powerful, and rechargeable battery has thoroughly changed our daily lives.^[^ ^2^ ^]^

In recent years, the rapid development of electric vehicles (EVs) has motivated the technical innovation of LIB industry.^[^ ^3^ ^]^ However, the implementation of LIBs in EVs to replace traditional internal combustion engine technology still faces great challenges in terms of energy density and charging time. For instance, the driving mileage of Tesla Model 3 is about 500 km with a fully charging time of more than 1 h by using a specific quick charger according to its owner's manual, while the conventional petrol‐driven cars can be refueled within 5 min with a longer driving mileage of above 750 km. Therefore, developing hybrid electric vehicles (HEVs) may be a compromising solution, which is more acceptable and economically more practical. Generally, high‐power batteries are required for HEVs to continually capture/release energy during braking, starting, and accelerating the vehicle in a short period of time.^[^ ^4^ ^]^ Unfortunately, the commonly used graphite anodes fail to meet the requirements for high‐power LIBs in HEVs owing to their insufficient electrochemical kinetics stemming from the sluggish Li‐ion diffusion rate.^[^ ^5^ ^]^ Recently, Wadsley–Roth crystallographic shear structure niobium‐based oxides including Nb_12_O_29_,^[^ ^6^ ^]^ TiNb_2_O_7_,^[^ ^7^ ^]^ Ti_2_Nb_10_O_29_,^[^ ^8^ ^]^ TiNb_24_O_62_,^[^ ^9^ ^]^ Nb_16_W_5_O_55_,^[^ ^10^ ^]^ Nb_18_W_8_O_69_,^[^ ^11^ ^]^ PNb_9_O_25_,^[^ ^12^ ^]^ etc., have been extensively investigated as high‐rate anode materials for LIBs. Their unique features of 3D open crystalline structure and reversible redox couples hold the promise of developing next‐generation LIBs characterized with high power density and long lifespan.

Besides, safety feature has always been a key design consideration for the practical application of LIBs, particularly high‐power ones.^[^ ^13^ ^]^ Moli Energy's first attempt to commercialize rechargeable Li‐based batteries with metallic Li as the anode failed due to serious safety hazards resulting from the uncontrolled growth of Li dendrites during cycling, forcing them to withdraw the product from the market.^[^ ^14^, ^15^ ^]^ One of the Nobel Laureates mentioned above, Akira Yoshino proposed replacing reactive Li in the anode with carbon‐based materials, which later became the foundation of commercially viable LIBs.^[^ ^16^ ^]^ In a fully charged LiMO_2_//graphite battery, the lithiated graphite anode (LiC_6_) possesses similar chemical reactivity to metallic Li.^[^ ^15^ ^]^ Thus, the solid electrolyte interface (SEI) play an important role in stabilizing the electrode/electrolyte interphase and constraining the continuous reaction between LiC_6_ and electrolyte while using graphite as the anode.^[^ ^17^ ^]^ However, the decomposition temperature of SEI was identified to be as low as 60 °C, which would trigger chain‐reactions between LiC_6_ and electrolyte, leading to the thermal runaway potentially.^[^ ^18^ ^]^ Moreover, lithium plating issue of the graphite anode during high‐rate cycling is also considered as an important incentive for easier thermal runaway or even the internal short circuit of batteries, due to its relatively low lithiation potential (<0.2 V vs Li^+^/Li).^[^ ^19^ ^]^ However, Li‐ion insertion into Wadsley–Roth crystallographic shear structure niobium‐based oxides mainly occurs above 1.0 V (vs Li^+^/Li), where the formation of SEI film is not necessary. Therefore, Wadsley–Roth phase anode materials can afford great advantages over the conventional graphite anode in terms of rate capability and safety performance, which should be promising candidates for high‐power LIB anodes in HEVs. Despite the outstanding characteristics of Wadsley–Roth crystallographic shear structure niobium‐based oxides, there is still extensive advancement space for further enhancing their electrochemical kinetics. And the industrial feasibility of Wadsley–Roth crystallographic shear structure niobium‐based oxides as anode materials for LIBs requires more systematic research.

This article is mainly concerned about how to realize the practical applications of Wadsley–Roth phase anode materials for high‐rate and high‐safety LIBs. The rest of the manuscript is organized as follows. Section 2 explains the inherent requirements for high‐safety LIB anodes and the crystal structure of Wadsley–Roth phase anode materials. Section 3 illustrates the electrochemical reaction mechanisms of Wadsley–Roth phase anode materials. Section 4 summarizes the modification strategies for the materials. Section 5 shows perspectives on the progress in constructing full cells based on Wadsley–Roth phase anodes. The last section presents the conclusions and outlook for the further development of Wadsley–Roth phase anode materials.

## 2 Preliminary Considerations

**Figure 1:**
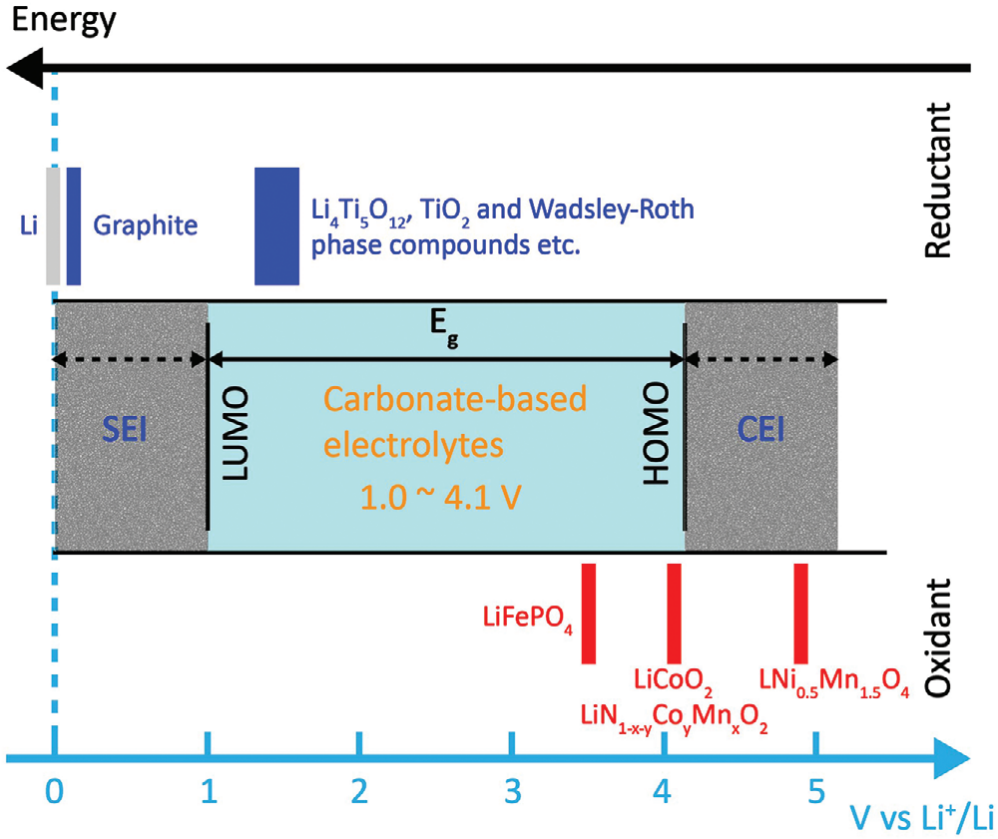
Energy and potential diagram illustrating the stability window of carbonate‐based electrolytes and the redox potentials of most cathodic and anodic materials applied in contemporary LIBs. *E_g_* represents the stability window of carbonate‐based electrolytes. For the cases of *μ*_A_>LUMO or *μ*_C_<HUMO, the formation of an SEI film is needed to maintain kinetic stability.

**Figure** **1** is a schematic of the relative electron energies and redox potentials of most cathode and anode materials applied in contemporary LIBs. Generally, an aprotic salt solution with a carbonate‐based organic solvent is the most widely used electrolyte, and the energy separation *E*_g_ of the highest occupied molecular orbital (HOMO) and lowest unoccupied molecular orbital (LUMO) of the electrolyte is accepted as its “stability window” (1.0–4.1 V vs Li^+^/Li). If the electrochemical potential at the anode (*μ*_A_) is above the LUMO energy level, the electrolyte will be reduced. Similarly, if the electrochemical potential at the cathode (*μ*_C_) is below the HOMO energy level, the electrolyte will be oxidized.^[^ ^20^ ^]^ Therefore, to ensure that the batteries are thermodynamically stable, *μ*_A_ and *μ*_C_ must be within the stability window of the electrolyte. A passivating SEI film can provide an extended window (≈5.0 V) through kinetic protection, allowing the graphite electrode to be used as the anode.^[^ ^21^ ^]^ However, at high charge rates, especially under extreme conditions, polarizations (electrochemical, concentration, and Ohmic) on the graphite electrode can increase the propensity of the SEI to break down and drive the anode potential to below the Li^+^/Li equilibrium potential. These negative effects lead to the formation of dendritic Li, which penetrates the separator and results in the short‐circuiting of the cells. To overcome the disadvantages of graphite anodes and realize a better rate capability and safety characteristic, the development of high‐performance anode candidates is essential.

The key for developing high‐safety anode materials is identifying ideal redox couples with moderate lithiation potential (*μ*_A_ > 1.0 V vs Li^+^/Li), to eliminate electrolyte decomposition and side reactions at a fundamental level. Thus, Li_4_Ti_5_O_12_, TiO_2_, and other Ti‐based oxides based on the redox couple Ti^4+^/Ti^3+^ are emerging as new anode materials for high‐performance LIBs.^[^ ^22^ ^]^ Among these, Li_4_Ti_5_O_12_ has been investigated extensively for the small volume change that occurs during the electrochemical Li intercalation/deintercalation process (0.2%) and the safe lithiation voltage (≈1.55 V vs Li^+^/Li).^[^ ^23^ ^]^ However, the relatively low theoretical specific capacity (175 mAh g^−1^) and the inherently low electronic conductivity restrict its rate performance. On the other hand, recently reported Wadsley–Roth phase compounds in the Nb_2_O_5_‐TiO_2_ and Nb_2_O_5_‐WO_3_ phase diagrams are some of the most promising families of anode materials for high‐safety LIBs, on the account of their open host structure for rapid Li^+^ diffusion and uninterrupted band nature for fast electron transport. Moreover, the two‐electron transfer per Nb atom (Nb^5+^/Nb^4+^ and Nb^4+^/Nb^3+^ redox couples) contributes to a high theoretical capacity and appropriate lithiation potentials in the range of 1.0–2.0 V (vs Li^+^/Li). **Table** **1** shows the summary of block type, the number of electrons transferred per formula unit (*n*), calcuated density, maximum theroretical gravimetric (volumetic) capacity for related Wadsley–Roth phase niobium‐based oxides in this review. It is obvious that the theoretical gravimetric capacities of Wadsley–Roth phase anode materials are much higher than that of Li_4_Ti_5_O_12_ (175 mAh g^−1^) due to the multi‐electrons transfer characteristic. Importantly, the theoretical volumetric capacity of Li_4_Ti_5_O_12_ is calculated to be only 592.2 mAh cm^−3^ based on the calculated density of 3.384 g cm^−3^. However, the theoretical volumetric capacities of these Wadsley–Roth phase compounds are above 1600 mAh cm^−3^. Therefore, Wadsley–Roth phase anode materials can be powerful substitutes for Li_4_Ti_5_O_12_ in high‐power LIB markets.

**Table 1:** Summary of block type, the number of electrons transferred per formula unit (*n*), calcuated density, maximum theroretical gravimetric (volumetic) capacity for various Wadsley–Roth phase compounds in this review. Where *n* is calculated based on the redox couples of Nb^5+^/Nb^3+^, Ti^4+^/Ti^3+^, W^6+^/W^4+^, V^5+^/V^3+^, Fe^3+^/Fe ^2+^, and Cr^3+^/Cr^2+^, the calculated density is obtained from the PDF‐4 database, and the maximum theoretical gravimetric capacity is calculated by the equation (*Q*_theoretical_ = *nF*/3.6*M*, *F* is Faraday's constant, and *M* is the molar mass)

Materials	Block type	*n*	Calculated density [g cm^−3^]	Maximum theoretical gravimetric capacity [mAh g^−1^]	Maximum theoretical volumetric capacity [mAh cm^−3^]
H‐Nb_2_O_5_	[(3 × 4)_1_ + (3 × 5)_∞_]	4	4.387	403.3	1769.2
TiNb_2_O_7_	(3 × 3)_∞_	5	4.328	387.6	1677.5
Nb_12_O_29_	(4 × 3)_∞_	22	4.567	373.4	1705.3
Ti_2_Nb_10_O_29_	(4 × 3)_∞_	22	4.541	396.0	1798.2
FeNb_11_O_29_	(4 × 3)_∞_	23	4.522	399.8	1807.9
AlNb_11_O_29_	(4 × 3)_∞_	22	4.483	389.7	1747.0
CrNb_11_O_29_	(4 × 3)_∞_	23	4.523	400.8	1812.9
GaNb_11_O_29_	(4 × 3)_∞_	22	4.646	379.0	1760.8
Nb_12_WO_33_	(3 × 4)_∞_	26	4.77	381.5	1819.8
Nb_14_W_3_O_44_	(4 × 4)_∞_	34	5.038	356.5	1796.0
Nb_16_W_5_O_55_	(4 × 5)_∞_	42	5.168	342.6	1770.6
Nb_18_W_8_O_69_	(5 × 5)_∞_	52	5.359	328.2	1758.8
TiNb_24_O_62_	(3 × 4)_2_	49	4.535	401.7	1821.7
PNb_9_O_25_	(3 × 3)_∞_	18	4.517	380.7	1719.6
VNb_9_O_25_	(3 × 3)_∞_	20	4.528	416.5	1885.9

**Figure 2:**
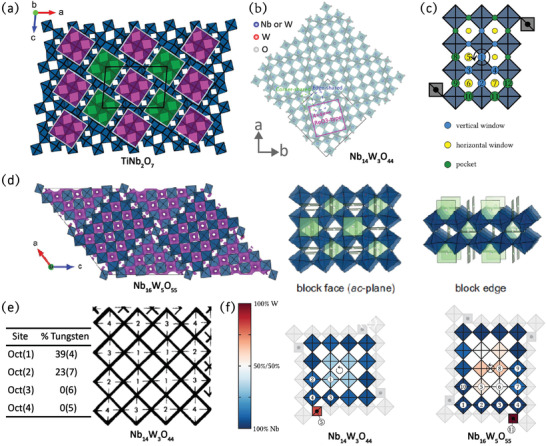
a) Crystal structure of TiNb_2_O_7_. Reproduced with permission.^[^ ^26^ ^]^ Copyright 2019, American Chemical Society. b) Crystal structure of Nb_14_W_3_O_44_. Reproduced with permission.^[^ ^27^ ^]^ Copyright 2020, Wiley‐VCH. c) Schematic diagram of three types of available Li^+^ sites in Nb_12_WO_33_. Reproduced with permission.^[^ ^28^ ^]^ Copyright 2019, American Chemical Society. d) Bond valence sum (BVS) maps and possible Li^+^ pathways in the Wadsley–Roth‐phase Nb_16_W_5_O_55_. Reproduced with permission.^[^ ^10^ ^]^ Copyright 2018, Springer Nature. e) The distribution of W over the octahedra sites. Reproduced with permission.^[^ ^29^ ^]^ Copyright 1983, The Royal Society of Chemistry. f) Cation occupancies of Nb and W atoms in the Wadsley–Roth‐phase Nb_14_W_3_O_44_ and Nb_16_W_5_O_55_. Reproduced with permission.^[^ ^28^ ^]^ Copyright 2019, American Chemical Society.

Wadsley–Roth phases generally occur in the chemical systems of Nb_2_O_5_‐NbO_2_, Nb_2_O_5_‐TiO_2_, and Nb_2_O_5_‐WO_3_.^[^ ^24^ ^]^ The basic structural units are blocks consisting of distorted MO_6_ octahedra sharing corners. The blocks are infinite in one dimension, and thus the structures are built of *n* × *m* × *∞* ReO_3_ type units, where *n* and *m* are the numbers of MO_6_ octahedra along the length and width of the blocks, respectively.^[^ ^25^ ^]^ The smallest known blocks in the Nb oxides correspond to 3 × 3 octahedra and the largest to 5 × 5 octahedra. The adjacent blocks are joined either through edge‐sharing or a combination of edge‐sharing and tetrahedrally coordinated metal atoms at the block corners. For example, TiNb_2_O_7_ comprises (3 × 3)_∞_ blocks, which are connected by edge‐sharing type only (**Figure** **2**a).^[^ ^26^ ^]^ While the structural units of Nb_14_W_3_O_44_ are (4 × 4)_∞_ ReO_3_‐type blocks, which are joined within the same *a*–*b* plane by corn‐sharing tetrahedra and to blocks offset along the *c*‐axis by edge‐sharing crystallographic shear planes (Figure 2b).^[^ ^27^ ^]^ Small differences in stoichiometry—varied from MO_3_ to MO_2_—can result in a significantly different degree of edge‐sharing. The MO_6_ octahedra cannot exclusively share corners, and hence an increasing number of edge‐shared octahedra are introduced.

There are several unique structural merits to the Wadsley–Roth phases that may facilitate rapid Li^+^ storage:

i) Kocer et al. demonstrated that there are three types of lithium sites in shear structures by density functional theory (DFT) calculations: vertical window positions, horizontal window positions, and pocket positions (Figure 2c).^[^ ^28^ ^]^ Abundant lithium sites in the Wadsley–Roth phases provide considerable active sites for Li^+^ intercalation and ensure a relatively high theoretical specific capacity.
ii) Griffith et al. investigated the bond valence sum (BVS) maps and possible Li pathways in the Wadsley–Roth‐phase Nb_16_W_5_O_55_ through bond valence energy landscape calculations (Figure 2d).^[^ ^10^ ^]^ The results revealed that Li^+^ migration is available not only in parallel tunnels of the block but also by hoops between the windows sites in the block interior. The 3D interconnected tunnels act as analogical multi‐lane highways for facile diffusion Li^+^ ions, which can effectively relieve the channel‐blocking issues at high rates.
iii) For some binary metal oxides with Wadsley–Roth phases, two types of MO_6_ octahedron are partially disorderly distributed in the block. Partial cation order in Nb_14_W_3_O_44_ was observed by Cheetham and Allen through neutron diffraction characterizations (Figure 2e).^[^ ^29^ ^]^ They found that W atoms prefer the center of the blocks, while Nb atoms prefer the corner sites due to the electrostatic interactions. The recent DFT studies of Nb_14_W_3_O_44_ and Nb_16_W_5_O_55_ further reinforced this perspective Figure 2f.^[^ ^28^ ^]^ The partial cation‐disorder effect in the block structure may prevent rate‐inhibiting long‐range Li ordering, which is beneficial for high‐rate cycling.^[^ ^10^ ^]^

## 3 Electrochemical Reaction Mechanisms

### 3.1 H‐Nb_2_O_5_

**Figure 3:**
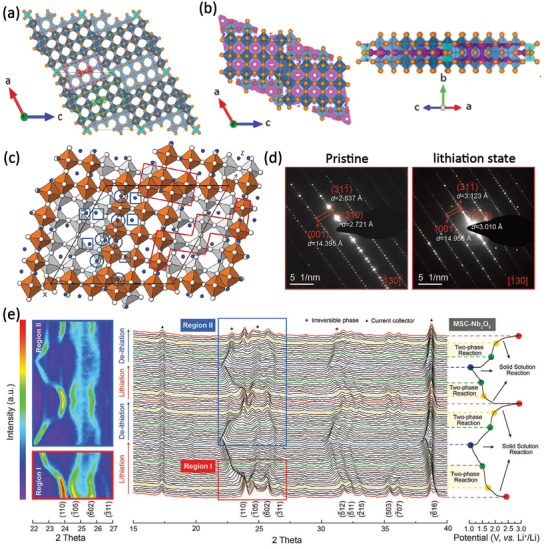
a) Crystal structure and b) BVS maps of H‐Nb_2_O_5_. Reproduced with permission.^[^ ^30^ ^]^ Copyright 2016, American Chemical Society. c) Crystalline structure of Li_1.714_Nb_2_O_5_ projection onto the (010) plane, from Rietveld refinement of neutron diffraction data. Fivefold and fourfold coordinated Li atoms are shown as circles and squares, respectively. Reproduced with permission.^[^ ^31^ ^]^ Copyright 2014, The Royal Society of Chemistry. d) The operando selected area electron diffraction patterns and e) operando XRD measurements accompanied by galvanostatic charge/discharge profiles of MSC‐Nb_2_O_5_. Reproduced with permission.^[^ ^32^ ^]^ Copyright 2020, Wiley‐VCH.

Monoclinic H‐Nb_2_O_5_ with a space group of *C*2/*m* (#12) belongs to the Wadsley–Roth family of crystallographic shear structures consisting of (3 × 4)_1_ and (3 × 5)_∞_ ReO_3_‐type blocks, denoted as [(3 × 4)_1_ + (3 × 5)_∞_] (**Figure** **3**a). The blocks extend infinitely along the *b‐*direction, and 1 and ∞ represent the block connectivity on the *a–c* plane. The BVS map of H‐Nb_2_O_5_ (Figure 3b) shows that there are extensive spaces within the blocks where Li^+^ ions could be accommodated.^[^ ^30^ ^]^ However, an obvious disruption can be observed on the iso‐surface, implying a relatively high diffusion energy barrier between the 3 × 5 and 3 × 4 blocks. The neutron powder diffraction measurements of chemically lithiated Li_1.714_Nb_2_O_5_ reveal two types of Li coordination in H‐Nb_2_O_5_: tetracoordinated perovskite‐type LiO_4_ (four‐coordinated) within the ReO_3_ blocks and LiO_5_ (five‐coordinated) square‐pyramidal sites on the block edges (Figure 3c).^[^ ^31^ ^]^ There are six LiO_4_ and LiO_5_ per asymmetric unit in the lithiated Li_1.714_Nb_2_O_5_ phase, which are denoted as circles and squares, respectively. An approximate volume change of 5.3% occurred during the Li^+^ insertion/extraction. Notable, the expansion of lattice parameter *b* was +8.1%. Nevertheless, the lattice parameters of *a* and *c* shrink by −1.7% and −1.2%, respectively, revealing high anisotropic lattice variation during Li‐(de)intercalation. The evolution of the lattice during the Li^+^ intercalation process can also be visualized by operando transmission electron microscopy images (Figure 3d). The lattice planes of (310) and (31−1) increase dramatically from 0.2739 to 0.3968 nm and from 0.2865 to 0.4210 nm, respectively, which further verifies the insertion of Li^+^ mainly along the *b*‐axis.^[^ ^32^ ^]^ Song et al. also studied the structural evolution of H‐Nb_2_O_5_ using in situ X‐ray diffraction (XRD) analysis, as shown in Figure 3e.^[^ ^32^ ^]^ H‐Nb_2_O_5_ is lithiated via two routes during the Li^+^ intercalation/extraction processes, in the voltage range of 1–3 V (vs Li^+^/Li): a two‐phase reaction corresponding to the flat regions of the charge/discharge profiles; and a solid–solution reaction corresponding to the slope regions. During the lithiation process, a slight right shift is observed in the crystalline planes parallel to the *b*‐axis, and an obvious left shift is observed in the crystalline planes of (110), (−311), (−511), (−215), and (−616). The expansion of the structure along the *b*‐axis and the minor crystal contraction along both the *a* and *c* planes are corroborated by the results.

In addition to H‐Nb_2_O_5_, there are also other polymorphs of Nb_2_O_5_ such as T‐Nb_2_O_5_, TT‐Nb_2_O_5_, and B‐Nb_2_O_5_, which do not belong to Wadsley–Roth phases. As shown in Figure S1 in the Supporting Information, the crystalline structure of T‐Nb_2_O_5_ is similar to that of tungsten bronzes but composed of highly distorted NbO_6_ octahedral and NbO_7_ pentagonal bipyramidal rather than regular octahedra.^[^ ^30^, ^33^ ^]^ TT‐Nb_2_O_5_ can be described as a disordering modification of T‐Nb_2_O_5_. The B‐Nb_2_O_5_ phase displays a TiO_2_(B)‐like structure. Among these Nb_2_O_5_ polymorphs, T‐Nb_2_O_5_ usually exhibits superior rate capability, owing to the fast Li^+^ intercalation pseudocapacitance and high Li^+^ diffusion rate in its room‐and‐pillar layered structure.^[^ ^34^ ^]^

### 3.2 TiNb_2_O_7_

**Figure 4:**
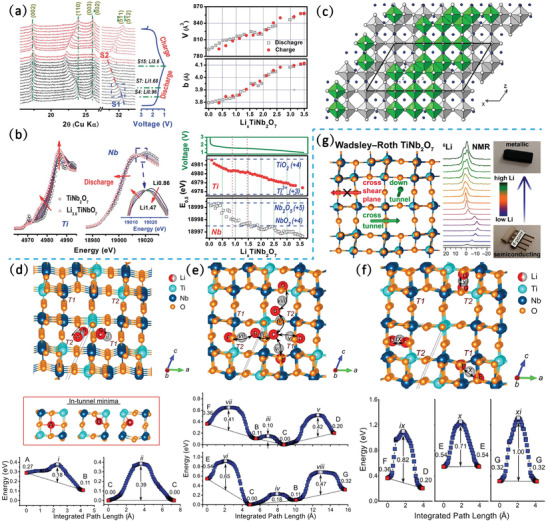
a) In situ XRD patterns and corresponding variation of lattice parameters b) In situ Ti K‐edge and Nb K‐edge XANES spectra of TiNb_2_O_7_ during the initial cycle at 0.1 C. Reproduced with permission.^[^ ^36a^ ^]^ Copyright 2014, The Royal Society of Chemistry. c) Crystal structure of Li_3.33_TiNb_2_O_7_ projection onto the (010) plane, from the Rietveld refinement of neutron diffraction data. Li atoms are represented as dark circles. Reproduced with permission.^[^ ^37^ ^]^ Copyright 2015, Elsevier. Li diffusion paths and corresponding energy barriers in TiNb_2_O_7_ d–e) within the blocks and f) across the blocks. g) Schematic diagram of three different types of Li conduction paths in TiNb_2_O_7_, and Li NMR paramagnetic shift corresponding to different amounts of Li in Li*_x_*TiNb_2_O_7_. Reproduced with permission.^[^ ^26^ ^]^ Copyright 2019, American Chemical Society.

The crystallographic structure of monoclinic TiNb_2_O_7_ with a space group of *C2/m* is built from (3 × 3)_∞_ blocks along the *b*‐axis (Figure 2a). Cheetham et al. found that the distribution of Ti and Nb in TiNb_2_O_7_ is not a completely random manner, but the Ti occupancy in the center of the block is 14.0% while that in the corner site is 64.5% through neutron diffraction experiments.^[^ ^35^ ^]^ A high theoretical specific capacity of 387.6 mAh g^−1^ is expected, based on the reductions of Nb^5+^ to Nb^3+^ and Ti^4+^ to Ti^3+^. The lithiation process of TiNb_2_O_7_ has been confirmed to be a complex, three‐stage intercalation mechanism that correlated with the amount of Li in Li*_x_*TiNb_2_O_7_ by in situ XRD (Figure **4**a).^[^ ^36^ ^]^ The first solid–solution reaction occurs from a pristine state to Li_1.0_TiNb_2_O_7_, while the second solid–solution reaction is observed during the evolution of Li_1.75_TiNb_2_O_7_ into its final form of Li_3.6_TiNb_2_O_7_. The region of two‐phase co‐existence corresponding to the evolution of Li_1.0_TiNb_2_O_7_ to Li_1.75_TiNb_2_O_7_ is evidenced by the apparent peak broadening, which is also reflected by the small voltage plateau at ≈1.5 V during the lithiation. The variation of the lattice parameter caused by Li^+^ insertion is highly anisotropic. During the discharging, lattice parameters *a* and *c* exhibit a trendless variation. However, the lattice parameter *b* and unit cell volume *V* exhibit a linearly increasing trend with the increase of the degree of lithiation, which is also in agreement with the XRD and powder neutron diffraction measurements of chemically lithiated Li*_x_*TiNb_2_O_7_ samples.^[^ ^37^ ^]^ This phenomenon may be attributed to the accumulation of Li^+^ ions along the tunnels of the block, which is in accordance with the direction of the *b*‐axis. The estimated unit‐cell volume change during the Li^+^ intercalation/extraction is ≈7.22–8.4%. The X‐ray absorption near‐edge spectroscopy (XANES) and X‐ray absorption fine structure results show simultaneous and equivalent changes in the charge compensation of Nb and Ti with the increase of the degree of lithiation in Li*_x_*TiNb_2_O_7_ (Figure **4**b). The valences of Nb and Ti are +3.6 and +3.2, respectively, with a cut‐off discharge voltage of 1.0 V.^[^ ^38^ ^]^

As shown in **Figure** **4**c, the Li coordination of TiNb_2_O_7_ can be also classified into two types, LiO_4_ and LiO_5_, and the numbers are in the ratio of 1:1. It is worth noting that the two types of active sites exhibit quite similar Li‐occupation tendencies, leading to the random distribution of Li^+^ ions over the available sites. Griffith et al. investigated the ionic and electronic conductions in TiNb_2_O_7_ through a series of experimental and computational analyses.^[^ ^39^ ^]^ The possible diffusion path of Li^+^ hopping between adjacent sites is evaluated by the activation energy (Figure 4d–f). The activation energies for Li_A_‐Li_B_ diffusion and Li_C_‐Li_C_ diffusions within the tunnel are 0.39 and 0.11 eV, respectively. The apparently low energy barriers suggest facile Li^+^ diffusion along the *b*‐axis (Figure 4d). The phenomenon of Li^+^ hopping can also occur between adjacent tunnels, which is supported by the small activation energies for the *a* and *c* axes diffusions (0.10 and 0.18 eV). However, larger energy barriers (0.71–1.00 eV) have been found in the crossblock diffusion, indicating the kinetic hindering of Li^+^ diffusion perpendicular to the tunnels near the crystallographic shear planes.

Briefly, facile Li^+^ diffusion was observed within the tunnels along the *b*‐axis and between the neighboring channels (Figure 4g). The crossblock diffusion, however, did not occur in any significant amount because of the high electrostatic repulsion near the crystallographic shear planes. The results also revealed that the *n*‐type doping effect of TiNb_2_O_7_ after the Li insertion increased the electronic conductivity by several orders of magnitude, this provides a deeper insight into the construction of the conductive network of the TiNb_2_O_7_ electrodes.^[^ ^26^ ^]^

### 3.3 Nb_12_O_29_ and Its Analogies

**Figure 5:**
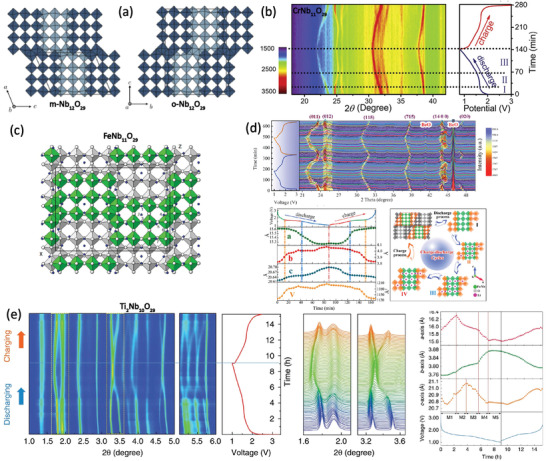
a) Crystal structures of m‐Nb_12_O_29_ and o‐Nb_12_O_29_. Reproduced with permission.^[^ ^41^ ^]^ Copyright 2019, American Physical Society. b) In situ XRD patterns of CrNb_11_O_29_ during the initial cycle at 0.4 C. Reproduced with permission.^[^ ^42c^ ^]^ Copyright 2019, American Chemical Society. c) Crystal structure of Li_11_FeNb_11_O_29_ projection onto the (010) plane, from Rietveld refinement of neutron diffraction data. Li atoms are represented as dark circles. Reproduced with permission.^[^ ^43^ ^]^ Copyright 2014, American Chemical Society. In situ XRD patterns and the corresponding variation of the lattice parameters of d) FeNb_11_O_29_ and e) Ti_2_Nb_10_O_29_ in the voltage window of 1.0–3.0 V. d) Reproduced with permission.^[^ ^44^ ^]^ Copyright 2019, Elsevier. e) Reproduced with permission.^[45]^ Copyright 2020, Springer Nature.

The basic crystalline structural units of Nb_12_O_29_ are (4 × 3)_∞_ blocks composed of corner‐shared NbO_6_ octahedra that infinitely extend along the *b*‐axis. A more exact expression of Nb_12_O_29_ is (Nb^4+^)_2_(Nb^5+^)_10_O_29_, with an average oxidation of 4.83, which means that there are two Nb^4+^(4d^1^) per 12 Nb sites. The resulting two unpaired electrons per block and shorter Nb–Nb distances endow Nb_12_O_29_ a metallic conductor with high electronic conductivity.^[^ ^40^ ^]^ According to different stacking forms, two similar Nb_12_O_29_ polymorphs of monoclinic *A2/m* and orthorhombic *Cmcm* structures are present (**Figure** **5**a).^[^ ^41^ ^]^ Goodenough group first reported that ≈9.5 Li^+^ could be intercalated/extracted into monoclinic Nb_12_O_29_, with a reversible capacity of ≈243 mAh g^−1^ at an extremely low current density of 0.0066 Ag^−1^.^[^ ^6^ ^]^ Since Nb^4+^ and Nb^5+^ are randomly distributed in the blocks, Nb^4+^ can also be substituted by other transition metals to form a series of Nb_12_O_29_ analogies with similar crystal structures, enhancing the cycling capacity (Ti_2_Nb_10_O_29_, CrNb_11_O_29_, FeNb_11_O_29_, AlNb_11_O_29_, GaNb_11_O_29_, etc.).^[^ ^42^ ^]^ Fu et al. prepared monoclinic CrNb_11_O_29_ through a thermal method and subsequent low‐temperature calcination. The introduction of conductive Cr^3+^ (t^3^_2g_e^0^_g_) ions with three free electrons in the 3d orbitals not only improves the conductivity, but also increases the unit‐cell volume, leading to a relatively high reversible capacity (401 mAh g^−1^).^[^ ^42c^ ^]^ The shift and recovery of characteristic peaks of CrNb_11_O_29_ in in situ XRD measurements indicate that the lithiation–delithiation processes in CrNb_11_O_29_ are based on a representative intercalation reaction mechanism (Figure 5b).

Another alternative Nb_12_O_29_ analogy is FeNb_11_O_29_, in which up to 23 electrons can be transferred per formula unit, due to the multiple redox couples of Fe^3+^/Fe^2+^ and Nb^5+^/Nb^4+^/Nb^3+^, which leads to an enhancement of the capacity up to 20% compared with that in case of Nb_12_O_29_. Neutron diffraction results of monoclinic FeNb_11_O_29_ demonstrated the complete disorderliness of Fe in the blocks. The crystalline structure of chemically lithiated Li_11_FeNb_11_O_29_ is shown in Figure 5c. Li atoms in Li_11_Fe_11_O_29_ are presented in six LiO_4_ and five LiO_5_ interactive modes, respectively, which are located within and between the 4 × 3 perovskite blocks of Fe(Nb)O_6_ octahedra.^[^ ^43^ ^]^ The volume change during the Li^+^ insertion/extraction is ≈6.5%. The volume expansion is anisotropic and mainly attributed to the expansion of the crystal along the *b*‐axis (+6.8%). In sharp contrast, the alteration of *c* is only +0.4%, and *a* even decreases by −1.1%. Zheng et al. also investigated the structural evolution of FeNb_11_O_29_ using in situ XRD characterization (Figure 5d).^[^ ^44^ ^]^ The lithiation process of FeNb_11_O_29_ in the voltage range of 1–3 V (vs Li^+^/Li) can be classified into four stages corresponding to two different lithiation mechanisms: I) The initial sloping region between 3.0 and 1.7 V is attributed to solid–solution reaction; II) between 1.70 and 1.58 V, a two‐phase transformation was observed during the formation of Li_3.29_FeNb_11_O_29_, induced by Li^+^ insertion into the 4i(1) and 4i(2) cavity sites; III) the continuous insertion of Li^+^ into the 4i(3) and 4i(4) cavity sites during the evolution of Li_3.29_FeNb_11_O_29_ into Li_8.34_FeNb_11_O_2_; IV) another solid–solution reaction region assigned to the last sloping region in the voltage range of 1.58–1.0 V resulting from the insertion of Li^+^ into the 4i(5) and 8j cavity sites during the evolution of Li_8.34_FeNb_11_O_2_ into Li_17.4_FeNb_11_O_2_. Through the Rietveld refinement of XRD data, the lattice volume expansion was estimated to be 6.92%, which is consistent with that estimated via neutron diffraction.

Ti_2_Nb_10_O_29_ is also a promising Nb_12_O_29_ analogy, and its theoretical capacity can go as high as 396 mAh g^−1^, based on the 24‐electron transfer. Similar to TiNb_2_O_7_, the partial cation order was also observed in Ti_2_Nb_10_O_29_ evidenced by power neutron diffractions, in which the Ti occupancy in the center octahedral sites of the block is 4.5%, while that in the corner octahedral sites is 64.5%.^[^ ^35^ ^]^ The lithiation mechanism of Ti_2_Nb_10_O_29_ was studied using in situ high‐energy synchrotron X‐ray diffraction (Figure 5e).^[^ ^45^ ^]^ The initial discharge–charge cycle witnessed drastic shifts in the Bragg peaks of Ti_2_Nb_10_O_29_, implying anisotropy in the lattice. As the amounts of lithiation increased, a four‐stage structural evolution process was observed corresponding to five monoclinic structures (denoted as M1–M5). Generally, the transformation from M1 to M4 is irreversible, resulting from the rearrangement of the Ti_2_Nb_10_O_29_ host to tolerate the insertion of Li^+^. During the delithiation process, the growth of the lattice parameter *b* is continuously inhibited, while *a/c* expands slightly, indicating that the Li^+^ diffusion and accumulation may have occurred along the *b*‐axis.

### 3.4 Niobium Tungsten Oxides

The existence of stable niobium tungsten oxides with the Wadsley–Roth phases in the Nb_2_O_5_‐WO_3_ binary‐phase diagram was first studied by Roth and Wadsley in 1965.^[^ ^46^ ^]^ Recently, several niobium tungsten oxides, such as Nb_18_W_8_O_66_, Nb_16_W_5_O_55_, Nb_14_W_3_O_44_, and Nb_12_WO_33_ have been extensively investigated as high‐safety anode materials for LIBs due to their unique inherent framework structures. In this section, we summarize some pioneering and representative studies about the lithiation mechanisms of the Wadsley–Roth phase niobium tungsten oxides.

#### 3.4.1 Nb_12_WO_33_

**Figure 6:**
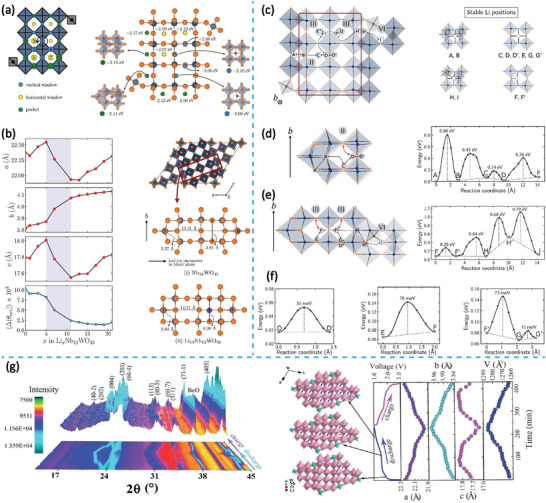
a) Schematic of three types of available Li^+^ sites in Nb_12_WO_33_. b) The lattice parameter changes of Nb_12_WO_33_ at different lithiation states and local structure in i) Nb_12_WO_33_ and ii) Li_13_Nb_12_WO_33_ along the second row of octahedra in the block obtained by DFT prediction. Reproduced with permission.^[^ ^28^ ^]^ Copyright 2019, American Chemical Society. c–f) Li diffusion paths and corresponding energy barriers in Nb_12_WO_33_. Reproduced with permission.^[^ ^47^ ^]^ Copyright 2020, American Chemical Society. g) In situ XRD patterns and corresponding variation of lattice parameters of Nb_12_WO_33_ during the initial galvanostatic discharge/charge cycle. Reproduced with permission.^[^ ^48^ ^]^ Copyright 2017, The Royal Society of Chemistry.

Monoclinic Nb_12_WO_33_ with the space group of *C2* belongs to Wadsley–Roth phases with (3 × 4)_∞_ ReO_3_ structure blocks. Unlike the structures of TiNb_2_O_7_ and Nb_12_O_29_ analogies in which the metal atoms are highly disordered, the crystalline structure of Nb_12_WO_33_ is highly ordered because all W atoms in Nb_12_WO_33_ are located at the tetrahedral positions at the junctions, with the remaining Nb atoms located at the octahedral sites (**Figure** **6**a). Koçer et al. systematically theorized the Li^+^ insertion mechanism and diffusion behavior of Nb_12_WO_33_.^[^ ^28^, ^47^ ^]^ Similar to other Wadsley–Roth phase materials, Li sites in Nb_12_WO_33_ can be broadly divided into two types, i.e., LiO_5_ (denoted as green circles) and LiO_4_ sites, respectively (Figure 6a). The LiO_4_ sites can be further classified into vertical “window” positions (denoted as blue circles) and horizontal “window” positions (denoted as yellow circles), depending on the direction of the window, with the planar block as reference.

The simulation result of a single Li atom in different active sites shows that Li atoms located at LiO_5_ sites (sites 8, 9, 10, 11, and 12) exhibit the lowest site energy, which is energetically more favorable. For the horizontal “window” positions (sites 5, 6, and 7), the Li atom may be located at the top of the plane created by four adjacent oxygen atoms with slightly higher site energies. The remaining vertical “window” positions may be too large for establishing the coordination between the Li and O atoms, and therefore, show the highest site energies. Generally, from the perspective of thermodynamics, the order in which lithium is inserted into the active sites of Nb_12_WO_33_ may be LiO_5_ sites, horizontal “window” LiO_4_ sites, and vertical “window” LiO_4_ sites.

A map of the structural evolution of Nb_12_WO_33_ during Li^+^ insertion can also be computed (Figure 6b). During the discharge process, the lattice parameter *b* exhibits a linearly increasing trend as the *x* in Li*_x_*Nb_12_WO_33_ increases, which is in agreement with the experimental results of in situ XRD (Figure 6g). However, lattice parameters *a* and *c* expand when 0<*x*<5, and then gradually shrink between *x* = 5 and *x* = 11. In the last lithiation region, *x* > 11, *a* and *c* increase again. The lattice expansion along the *b*‐axis and the lattice contraction along the *a* and *c* axes are probably related to the change of the local structure, which is in part reflected by the average octahedral distortion (denoted as Δ(*θ*_oct_)) and bond distance. Compared with pristine Nb_12_WO_33_, the block height of Li_13_Nb_12_WO_33_ increases from 0.381 to 0.409 nm, whereas the Nb–Nb distance along the shear plane decreases from 0.337 to 0.294 nm. The Li^+^ accumulation along the *b*‐axis and the decrease of bond distance along the *a* and *c* axes not only lead to the anisotropic evolution of the lattice parameters, but also improve the crystalline symmetry. This improvement is confirmed by the most conspicuous change of Δ(*θ*_oct_) occurs between *x* = 5 and *x* = 11, which exactly corresponds to the region of intense shrinkage observed for lattice parameters *a* and *c*.

The determination of the Li^+^ migration path is necessary for understanding the lithiation mechanism, the minimum energy paths and activation barriers (NEB) for Li^+^ motion in Nb_12_WO_33_ are summarized in Figure 6c–f. The ion diffusion behavior can be classified into i) diffusion perpendicular to the block plane (along the *b*‐axis) and ii) diffusion parallel to the block plane (along the *a–c* plane). The diffusion parallel to the block plane can be further divided into two types: intrablock and crossblock, respectively. The crossblock diffusion, either by migrating through cavity IV next to the W tetrahedral (H↔H′↔I) or by crossing the shear plane (A↔B), presents the highest energy barriers (0.79 and 0.80 eV, respectively).

For the intrablock diffusion along the *a–c* plane, the energy barrier needed for Li^+^ to hop over from LiO_5_ pocket sites at the block edges into the block center (B↔C and H↔F′ *E*_a_ = 0.44 eV) is relatively higher than that between the cavities within the same block (F↔F′ *E*_a_ = 0.2 eV, D↔F *E*_a_ = 0.36 eV). All Li^+^ hops along the *b*‐axis exhibit exceedingly low energy barriers (D↔D′ *E*_a_ = 0.035 eV, E↔F *E*_a_ = 0.07 eV, F′↔G′ *E*_a_ = 0.073 eV). Based on the above analysis, the Li^+^ diffusion features in Nb_12_WO_33_ can be summarized as follows: i) Li^+^ hopping between cavities perpendicular to the block plane has the lowest energy barrier and cover distances of 0.019 nm, which is half that in case of the lattice parameter *b*, revealing the rapid long‐range Li^+^ conduction along the *b*‐axis; ii) the Li^+^ hopping between the two tunnels in the center is much more facile than that between the two tunnels close to the edges; iii) the energetics of Li^+^ diffusion across the crystallographic shear planes of the contiguous blocks are greatly impeded.

#### 3.4.2 Nb_14_W_3_O_44_

In tetragonal Nb_14_W_3_O_44_, (4 × 4)_∞_ blocks on the same plane are connected by tetrahedrally coordinated W atoms. In separated planes, the blocks are joined along the *c*‐axis by crystallographic shear edge‐sharing.^[^ ^49^ ^]^ Unlike the ordered Nb_11_WO_33_ blocks, the Nb_14_W_3_O_44_ blocks are composed of residual WO_4_ and all NbO_4_ octahedra. The statistically distributed Nb and W atoms probably cause disorder among the cations. Recent studies have reported that the distributions of Nb and W atoms in the blocks were not entirely random, and that W atoms may be inclined to reside in block‐central sites within the block.

**Figure 7:**
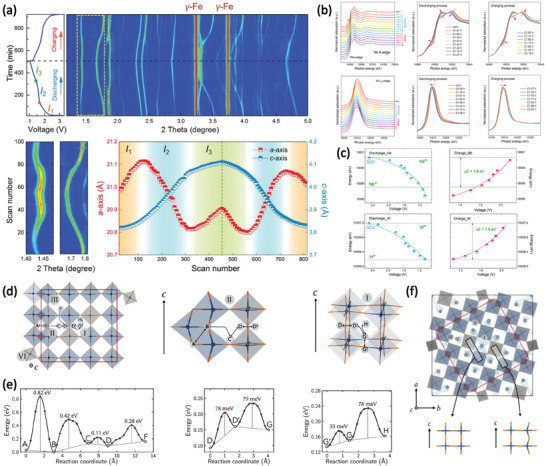
a) In situ synchrotron high‐energy XRD and the corresponding variation of the lattice parameters of Nb_14_W_3_O_33_ during the initial galvanostatic discharge/charge cycle. b) In situ XANES spectra and c) energy shifts of Nb‐K absorption edge and W‐L_3_ absorption edge as a function of voltage. Reproduced with permission.^[^ ^27^ ^]^ Copyright 2020, Wiley‐VCH. d,e) Li^+^ diffusion paths and the corresponding energy barriers in Nb_14_W_3_O_44_. f) Li^+^ probability density distribution within Li_5_Nb_14_W_3_O_44_ obtained from the AIMD simulations. Reproduced with permission.^[^ ^47^ ^]^ Copyright 2020, American Chemical Society.

In a previous study, we synthesized micro‐sized Nb_14_W_3_O_44_ with high crystallinity and studied its structural change and redox behavior during cycling using in situ synchrotron high‐energy XRD and in situ XANES (**Figure** **7**a–c).^[^ ^27^ ^]^ As shown in the contour plot of Figure 7a, the characteristic diffraction peaks of Nb_14_W_3_O_44_ exhibit a consistent reversible shift and recovery, without the appearance of a new peak, revealing a single‐phase solid–solution reaction mechanism. Similar to Nb_12_WO_33_, the evolution of lattice parameters in Nb_14_W_3_O_44_ during the Li^+^ intercalation/extraction process is anisotropic. The evolution can be divided into three basic regions. Both *a* and *c* expand during the initial discharge state (region *I*_1_) between 3.0 and 1.7 V (vs Li^+^/Li). In region *I*_2_, which corresponds to the voltage range of 1.7–1.3 V, *c* continues to expand, implying that the phenomenon of Li^+^ diffusion may be occurring mainly along the *c*‐axis. The contraction of *a* can be observed in this region as well, which can be attributed to the electrostatic attraction between the Li and O atoms. Subsequently, a reversal is observed in *a* as it increases again till the cut‐off voltage of 1.0 V in region *I*_3_ is reached, which may be related to the deep reduction of Nb^4+^/Nb^3+^ and W^5+^/W^4+^ redox couples verified by in situ XANES. O_2p_ may compensate for the additional charges of Nb^3+^ and W^4+^ via the highly hybridized Nb‐O and W‐O routes. Thus, the interaction between the O and Li atoms weakened with further lithiation.

In situ XANES reveal the mechanism of redox reaction occurring in the Nb_14_W_3_O_44_ electrode (Figure 7b,c). During the initial discharging process, the Nb *K*‐edge shifted gradually to a lower energy state, which is consistent with the expected contribution from the Nb^5+^/Nb^4+^ and Nb^4+^/Nb^3+^ redox capacity (Figure 7b). A similar red‐shift trend is observed for the W *L_3_*‐edge spectra. Thus, it can be rationally deduced that Nb^5+^ ions were partially reduced to Nb^3+^, while nearly all W^4+^ ions were reduced to W^3+^ during cycling (Figure 7c). Most importantly, both Nb *K*‐edge and W *L_3_*‐edge can recover to their original states with only a minor shift while charging up to 3.0 V, demonstrating the excellent reversibility of the redox couples.

The NEB for Li^+^ mobility in Nb_14_W_3_O_44_ was also performed (Figure 7d,e), and the results are similar to those in the case of Nb_12_WO_33_.^[^ ^47^ ^]^ The crossblock diffusion traversing the shear plane (A↔B) also presents the highest energy barriers (0.82 eV). Additionally, the energy barrier for Li^+^ hopping from the pocket site at the block edge into the window site (B↔C) is the second‐highest value (0.42 eV). Cross‐cavity diffusions along the *a–c* plane within the block have moderate energy barriers (D↔F *E*_a_ = 0.0.28 eV, C↔D↔D′↔G *E*_a_ = 0.07 eV). The lowest energy barriers can also be found for the case where Li^+^ hops perpendicular to the block plane (C↔D *E*_a_ = 0.011 eV, G↔D′ *E*_a_ = 0.033 eV, G↔H *E*_a_ = 0.078 eV). AIMD (Ab initio molecular dynamics) simulation of Li_8_Nb_14_W_3_O_44_ can help understand the dynamics of Li^+^ motion (Figure 7f). There is an interconnected migration network between the type I and type II cavities, which are not connected to the tunnels in the type III cavity located at the block edge. This is consistent with the NEB results.

#### 3.4.3 Nb_16_W_5_O_55_ and Nb_18_W_8_O_69_

**Figure 8:**
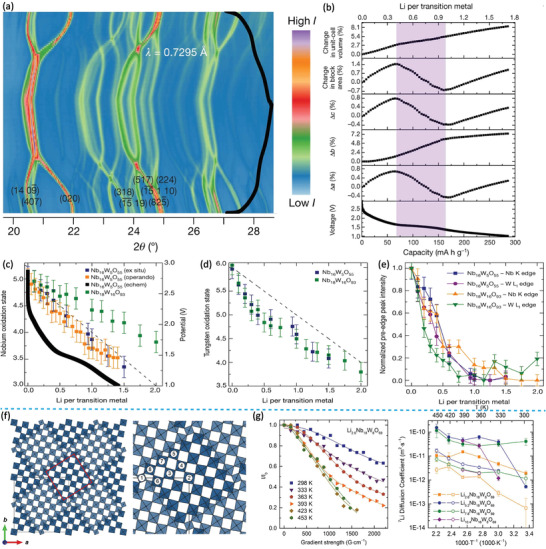
a) Operando synchrotron XRD and b) the corresponding variation of the lattice parameters of Nb_16_W_5_O_55_. Oxidation states of c) Nb and d) W and e) prestage integrated intensity as a function of lithiation obtained from operando and ex situ Nb K edge and W L_I, II_ XANES spectra. Reproduced with permission.^[^ ^10^ ^]^ Copyright 2018, Springer Nature. f) Crystal structure and metal cation sites of Nb_18_W_8_O_69_. g) Li diffusion pulsed field gradient NMR spectroscopy of Li*_x_*Nb_18_W_8_O_69_. Reproduced with permission.^[^ ^11^ ^]^ Copyright 2020, American Chemical Society.

Monoclinic Nb_16_W_5_O_55_ belongs to the Wadsley–Roth phase with (4 × 5)_∞_ ReO_3_ structure blocks (Figure 2c). As with those in Nb_14_W_3_O_44_, all tetrahedral sites in Nb_16_W_5_O_55_ are occupied by W atoms, and the blocks are composed of statistically distributed WO_4_ and NbO_4_ octahedra (Figure 2d). Griffith et al. investigated the structural evolution and redox activity of Nb_16_W_5_O_55_ through the operando synchrotron XRD (**Figure** **8**a,b).^[^ ^10^ ^]^ As shown in Figure 8a, the lithiation process of Nb_16_W_5_O_55_ is a three‐stage solid–solution mechanism. 1) First, the block expands along the *a–c* plane and slightly expands along the *b‐*axis, corresponding to the region of Nb_16_W_5_O_55_–Li_8.4_Nb_16_W_5_O_55_. 2) The block experiences anisotropic evolution involving a contraction along the *a–c* plane and a significant expansion perpendicular to the block plane, corresponding to the region of Li_8.4_Nb_16_W_5_O_55_–Li_21_Nb_16_W_5_O_55_. 3) All lattice parameters, *a*, *b*, and *c*, increase monotonically corresponding to the region beyond Li_21_Nb_16_W_5_O_55_. Moreover, the volume expansion is ≈5.5% at Li_21_Nb_16_W_5_O_55_ lithiation (Figure **8**b). The multielectron redox behavior of Nb_16_W_5_O_55_ during the Li^+^ insertion was also investigated by operando and ex situ XANES. With the increase of the lithiation degree, the oxidation state of Nb exhibits a linear shift from +5 to +3.5 (Figure 8c). The trend for W is similar to that of Nb (Figure 8d); however, its oxidation state shifts significantly in the region of Nb_16_W_5_O_55_–Li_10.5_Nb_16_W_5_O_55_. These results imply that W atoms may be more preferentially reduced than Nb atoms during the Li^+^ intercalation of Nb_16_W_5_O_55_. Because the reduction of d° cations of Nb^5+^ and W^6+^ ions decreases the second‐order Jahn–Teller distortions, the local symmetry of Li*_x_*Nb_16_W_5_O_55_ is also gradually improved as the amount of lithiation increases (Figure 8e).

Nb_18_W_8_O_69_ is made of 5 × 5 large blocks with an area of ≈2 × 2 nm^2^ (Figure 8f).^[^ ^11^ ^]^ Similar to other niobium tungsten oxides mentioned earlier, the M1 site in Nb_18_W_8_O_69_ is occupied by a WO_4_ tetrahedron. Although Nb^5+^ and W^6+^ are distributed randomly throughout the block, there is still a statistical trend. Electrostatic repulsion at the edge‐shared octahedra along the crystallographic shear planes gives rise to local cation ordering from the W^6+^ ions with a higher charge favoring the block center (M2–M4) and the Nb^5+^ cation with a lower charge favoring the block edges (M5–M8). The Li pulsed‐field gradient NMR spectra of Li*_x_*Nb_18_W_8_O_69_ were measured to determine its ionic transport properties (Figure 8g). The Li^+^ diffusion coefficients (*D*_Li+_) of Li*_x_*Nb_18_W_8_O_69_ reach 1 × 10^−13^ to 4 × 10^−11^ m^2^ s^−1^ at room temperature. At 80 °C, *D*_Li+_ increases to as high as 2 × 10^−10^ m^2^ s^−1^. The rapid Li^+^ diffusion rate at high temperatures may be related to the unique 5 × 5 large block structure with 16 parallel tunnels for long‐range Li^+^ migration.

### 3.5 TiNb_24_O_62_, PNb_9_O_25_, and VNb_9_O_25_

**Figure 9:**
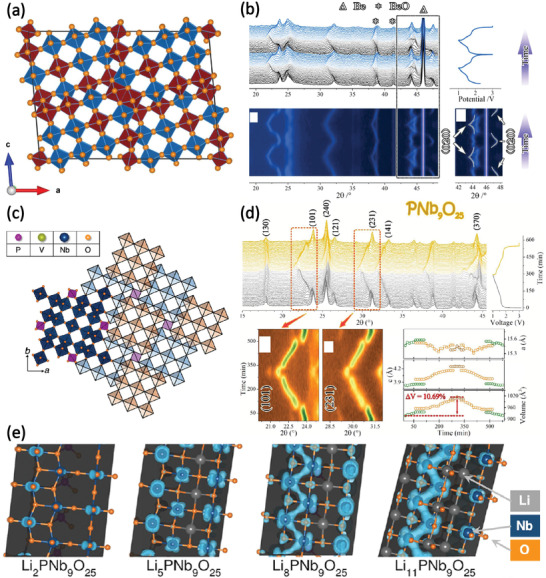
a) Crystal structure of TiNb_24_O_62_ in which the sites preferentially occupied by Ti polyhedral are shown in red and Nb‐rich octahedra are shown in blue. Reproduced with permission.^[^ ^9^ ^]^ Copyright 2017, American Chemical Society. b) In situ XRD patterns and corresponding contour plots of TiNb_24_O_62_ during cycling. Reproduced with permission.^[^ ^50^ ^]^ Copyright 2018, Elsevier. c) Crystal structure of PNb_9_O_25_. The different colors of the octahedral blocks on the right indicate their relative positions along the *c* direction. Reproduced with permission.^[^ ^52a^ ^]^ Copyright 2020, American Chemical Society. d) In situ XRD patterns of PNb_9_O_25_ and the variations of lattice parameters during cycling. Reproduced with permission.^[^ ^54^ ^]^ Copyright 2020, The Royal Society of Chemistry. e) Crystal structures of lithiation Li*_x_*PNb_9_O_25_ across various compositions and the corresponding d‐electron densities. Reproduced with permission.^[^ ^52a^ ^]^ Copyright 2020, American Chemical Society.

In addition to the extensively studied H‐Nb_2_O_5_, TiNb_2_O_7_, Nb_10_O_29_ and its analogies, and Nb‐W oxides, there are also some other Nb‐based oxides with the related Wadsley–Roth phase. TiNb_24_O_62_ also belongs to the Wadsley–Roth phase, which is consisted of 3 × 4 blocks split into pairs, described as (3 × 4)_2_ (**Figure** **9**a).^[^ ^9^ ^]^ Through the high‐resolution neutron power diffraction of TiNb_24_O_62_, Griffith et al. found the preference of Ti^4+^ over the higher‐valent Nb^5+^ in the tetrahedral metal site with the total occupancy of Nb and Ti atoms refined to 0.942 and 0.058, respectively, which are close to random values of 0.96 and 0.04. Yu et al. investigated the lithiation mechanism of TiNb_24_O_62_ by in situ XRD (Figure 9b).^[^ ^50^ ^]^ During discharging, the (020) peak of TiNb_24_O_62_ disappears and a new phase is formed at ≈44.13°, which can be assigned to the lithiated TiNb_24_O_62_ (Li_15.7_TiNb_24_O_62_) phase. And the occurrence of phase transformation during cycling was also verified by the galvanostatic intermittent titration technique measurement of TiNb_24_O_62_ by Griffith et al.^[^ ^9^ ^]^ The subsequent intercalation of Li^+^ ions further causes the shift of this peak to a lower *θ*. Finally, TiNb_24_O_62_ is fully lithiated to Li_28_TiNb_24_O_62_ with a volume expansion of 17.5%. Upon charging, all the reflection peaks including the (020) peak of TiNb_24_O_62_ recover to the initial positions. It should be noticed that the recent study about the entropy stabilization of TiO_2_‐Nb_2_O_5_ Wadsley–Roth shear phases indicated that TiNb_24_O_62_ may convert into energetically more stable phases during repeated cycling in batteries, which will affect the long‐term cycling stability.^[^ ^51^ ^]^

Tetragonal PNb_9_O_25_ with the space group of *I4/m* belongs to Wadsley–Roth phases with (3 × 3)_∞_ ReO_3_ structure blocks. As shown in Figure 9c, the crystalline structure of PNb_9_O_25_ is built up from nine NbO_6_ octahedra corner‐share to form a 3 × 3 block in the *ab* plane, and these blocks are held together by corner‐sharing PO_4_ tetrahedra.^[^ ^12^, ^52^ ^]^ It is interesting that the P atoms in PO_4_ tetrahedra can also be substituted by redox‐active V to obtain VNb_9_O_25_ with a similar crystal structure.^[^ ^53^ ^]^ The lithiation mechanism of PNb_9_O_25_ is a combination of three solid–solution regions (the sloping voltage profile between 3.0 and 1.62 V, 1.54 and 1.11 V, and 1.08 and 1.0 V) and two biphasic regions (two plateaus around 1.62 and 1.11 V), determined by in situ XRD (Figure 9d).^[^ ^54^ ^]^ And the expansion of lattice volume after full lithiation is estimated to be about 10.69%. Besides, DFT calculations of Nb partial densities of states for Li*_x_*PNb_9_O_25_ demonstrated that d orbital overlap between the edge‐sharing octahedra increases with the increasement of lithium composition (Figure 9e).^[^ ^52a^ ^]^ Such an insulator‐to‐metal transition in PNb_9_O_25_ during Li^+^ insertion may be beneficial to its high rate performance in microsized particles.

## 4 Strategies to Enhance the Electrochemical Performance

**Figure 10:**
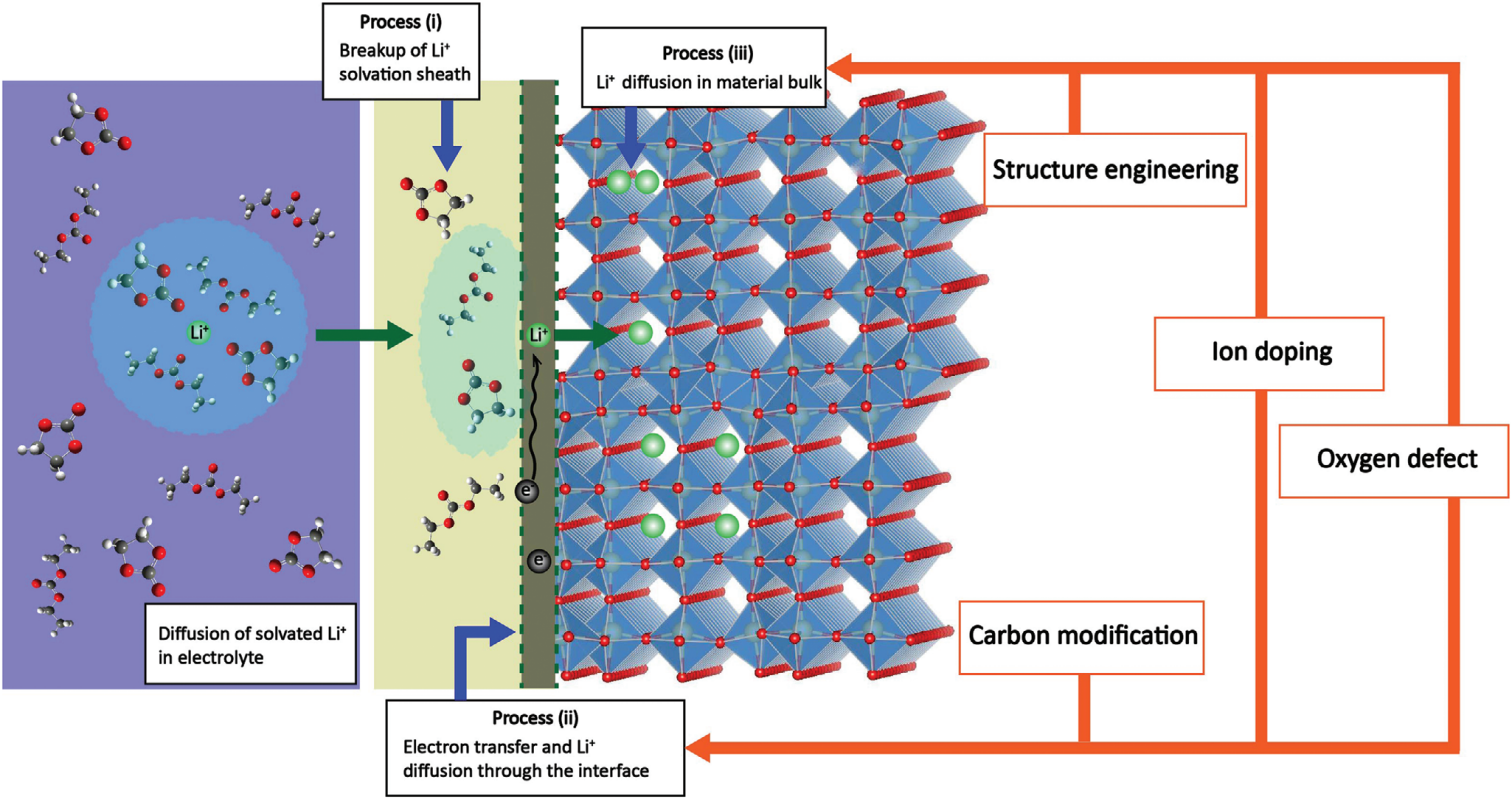
Schematic diagram of Li^+^ insertion into the anode and functions of different modification strategies.

Although Wadsley–Roth crystallographic shear structure anode materials have shown great potential for high‐rate Li^+^ storage, their rate performance and cycling stability still require further improvement due to their inherently low electronic conductivity. In addition, the apparent Li^+^ diffusion rate in an actual battery system is affected by the particle size and electrode architecture. From the perspective of electrochemistry, the process of Li^+^ injection into the anode can be divided into three processes (**Figure** **10**): i) Li^+^ ions pass through an electrical double layer and are liberated from the solvation sheath at the electrode/electrolyte interface; ii) the transfer of electrons and migration of de‐solvated Li^+^ across the interface; and iii) diffusion of Li^+^ into the bulk structure of the anodes. Because of the absence of an SEI film on the surface of the Wadsley–Roth phase anodes, the de‐solvation active energies of Li^+^ should be essentially determined by the nature of the electrolyte solvent and Li salts. Therefore, the enhancement electrochemical kinetics of Wadsley–Roth phase anode materials should mainly focus on the manipulation of (ii) and (iii). We analyzed the corresponding works published from 2017 to the most recent ones and present the following conclusions.

### 4.1 Structural Engineering

To enhance the high‐rate cycling performance of Wadsley–Roth phase anode materials, researchers have explored methods such as reducing the size of the anode and constructing the anode with a porous structure. Li^+^ diffusion in a host material (process iii) is not only determined by the intrinsic Li^+^ diffusion energy barriers but also by the diffusion length in the material, which can be represented as: $\tau \;=\frac{L^{2}}{D_{\mathrm{Li}}}\;$ (where *τ* is the characteristic time for ions to diffuse through the electrode material, *D*_Li_ is the intrinsic diffusion coefficient, and *L* is the diffusion length). Therefore, the reduction of particle size *L* should reduce the diffusion time energetically up to a certain extent.^[^ ^55^ ^]^ Furthermore, reduced particle size can also help alleviate the strain arising from volume variations, because the toughness and adhesion effects within the grain boundaries may increase at the nanoscale, which is favorable to the stabilization of the crystalline host during charge–discharge.^[^ ^56^ ^]^

The design of 1D nanostructure is a commonly investigated route to engineer Wadsley–Roth phase anode materials. One such design method, the electrospinning method, is used for the fabrication of nanofibers, nanowires, and nanotubes. Yan et al. fabricated Nb_14_W_3_O_44_ nanowires with a diameter of ≈400 nm by using the electrospinning method with stoichiometric amounts of ammonium metatungstate hydrate, niobium oxalate, nitric acid, and poly(vinyl pyrrolidone) (PVP) dissolved in a mixture of ethanol and deionized water as the electrospinning solution.^[^ ^57^ ^]^ The relatively high apparent Li^+^ diffusion coefficient of Nb_14_W_3_O_44_ nanowires (8.02 × 10^−13^ cm^2^ s^−1^), which is comparable with that of Li_4_Ti_5_O_12_, contributed to the high initial charge/discharge capacities of 215.9 and 264.7 mAh g^−1^, respectively. However, the cycling current density was limited to only 0.8 A g^−1^. AlNb_11_O_29_,^[^ ^42a^ ^]^ TiNb_24_O_62_,^[^ ^50^ ^]^ Nb_12_WO_33_,^[^ ^48^ ^]^ Ti_2_Nb_10_O_29_,^[^ ^58^ ^]^ and TiNb_2_O_7_ nanowires^[^ ^59^ ^]^ have also been synthesized by the electrospinning method, and their improved electrochemical performance compared with that of bulk materials has clearly demonstrated the influence of the nanowire architecture on the Li^+^ diffusion rate.

By further modifying the electrospinning parameters, hollow nanofibers and hollow nanotubes with larger surface areas can be also obtained.^[^ ^36^, ^44^, ^60^ ^]^ A representative sample is the 1D TiNb_2_O_7_ nanofibers synthesized by the one‐step dual‐nozzle coaxial co‐electrospinning method.^[^ ^36b^ ^]^ After calcination, the inner PVP and polyacrylonitrile polymer were removed, and hollow nanofibers with a wall thickness and outer thickness of ≈600 and 200 nm, respectively, were formed by connecting the nanosized TiNb_2_O_7_ particles along the orientation of the fiber. The hollow TiNb_2_O_7_ nanofibers exhibited smaller redox polarizations and better cycling stabilities than the TiNb_2_O_7_ nanofibers.

In addition to the aforementioned 1D nanostructures, porous structures provide another alternative for the structural modification of Wadsley–Roth phase anode materials without sacrificing much volumetric energy density.^[^ ^61^ ^]^ Lou et al. synthesized porous Ti_2_Nb_10_O_29_ nanospheres with a diameter of ≈300–500 nm through a facile one‐pot solvothermal method.^[^ ^62^ ^]^ The unique porous hierarchical architecture of the anode demonstrably enhanced the pseudocapacitive‐based charge storage, and a high capacity of 141.5 mAh g^−1^ can be retained after 1000 cycles at a high current density of 10 C. Ti_2_Nb_10_O_29_ with a crosslinked hierarchical porous structure was reportedly obtained using a versatile bioinspired solvothermal‐template method that uses biomass‐derived carbon as the sacrificial templates.^[^ ^63^ ^]^ A considerable capacity of 192 mAh g^−1^ can be maintained after 300 cycles at 1 C in the solid electrolyte.

### 4.2 Carbon Modification

The integration of Wadsley–Roth phase anode materials with carbon‐based material, such as amorphous carbon,^[^ ^64^ ^]^ graphene,^[^ ^65^ ^]^ carbon nanotubes (CNTs),^[^ ^66^ ^]^ and carbon nanofibers (CNFs),^[^ ^67^ ^]^ to form a hybrid structure has been proved to be an effective strategy for overcoming the intrinsically poor electronic conductivity of these materials. In particular, carbon modification and structural engineering approaches can be adopted simultaneously to combine the benefits of the synergetic optimization of processes (ii) and (iii) for fast charge storage.

Shen et al. used solvothermal electrodeposition (ST‐ED), to construct binder‐free vertical graphene (VG)/TiNb_2_O_7_@S‐C core/shell arrays.^[^ ^68^ ^]^ In this architecture, TiNb_2_O_7_ nanoparticles are sandwiched between the VG skeleton and an S‐C shell transformed from an electrodeposited poly(3,4‐ethylene dioxythiophene) layer, ensuring the continuous and percolating electrons/ion transport network. Moreover, the increased active electron density of the S‐doped carbon shell benefits the adsorption performance of Li^+^ ions, which is expected to improve the electrochemical performance further. Consequently, the VG/TiNb_2_O_7_@S‐C electrode exhibits a capacity of 193 mAh g^−1^ with a capacity retention of 78% after 5000 cycles at 10 C.

In the work of Song et al., an amorphous N‐doped carbon film is covered on the surface of micrometer‐sized single‐crystal H‐Nb_2_O_5_ (MSC‐Nb_2_O_5_) to obtain N‐C@MSC‐Nb_2_O_5_.^[^ ^32^ ^]^ The N‐doped carbon shell not only promotes the homogeneity of electron transport in the particles, but also eliminates the spatiotemporal asynchronization of the lithiation/delithiation processes caused by the local inhomogeneity of Li^+^ ions. The improved electrochemical reaction kinetics and reversibility endow the N‐C@MSC‐Nb_2_O_5_ with better cycling stability (170 mAh g^−1^ after 1000 cycles at 2 A g^−1^) than that of MSC‐Nb_2_O_5_ without carbon modification (only 65 mAh g^−1^). 1D CNFs or CNTs can also be used to form a conductive network for anode modification. Tang et al. combined a combination of electrophoretic deposition and solvothermal methods to grow hierarchical CNFs@Ti_2_Nb_10_O_29_ arrays on carbon cloth (CNFs@Ti_2_Nb_10_O_29_/CC).^[^ ^69^ ^]^ Ti_2_Nb_10_O_29_ particles with a size of 20–50 nm were tightly anchored to the intertwined and interconnected CNFs to form a whole conductive network. Moreover, the Li^+^ diffusion coefficients of CNFs@Ti_2_Nb_10_O_29_/CC can reach as high as 2.59 × 10^−11^ cm^2^ s^−1^, which is approximately two orders of magnitude larger than that of bulk Ti_2_Nb_10_O_29_ (1.45 × 10^−12^ cm^2^ s^−1^).

### 4.3 Ion Doping

Ion doping can optimize the electrochemical performance of battery materials by changing the lattice constants and band gap to improve the Li^+^ diffusion rate and electronic conductivity. For Wadsley–Roth phase anode materials, the main doping sites are cation sites. According to Yang et al., and more recently Deng et al.,^[^ ^70^ ^]^ Cr^3+^ doping by substituting Ti^4+^ in the crystalline structure of Ti_2_Nb_10_O_29_ has been reported to increase the conductivity to 2.80 × 10^−4^ S cm^−1^ by forming of impurity bands around the Fermi level, which is an astounding six orders of magnitude higher than that of an undoped one (≈1.61 × 10^−10^ S cm^−1^). A serendipitous advantage of this method is that Cr^3+^ doping increases the unit‐cell volume and decreases the particle size. The as‐prepared Cr_0.6_Ti_0.8_Nb_10.6_O_29_ sample, among different doping amounts of Cr^3+^ showed the best cycling stability with a capacity loss of only 0.01% per cycle over 500 cycles at 10 C. In addition, Cr^3+^ doping can be also introduced into the crystalline structures of FeNb_11_O_29_ and Nb_12_O_29_.^[^ ^71^ ^]^ Moreover, other dopants of Cu^2+^, Mn^2+^, and V^5+^ have been investigated and shown to be advantageous in improving the electrochemical kinetics of Wadsley–Roth phase anodic materials.^[^ ^72^ ^]^

### 4.4 Oxygen Vacancies

Recently, defect engineering has emerged as a powerful technology to impart features such as more active sites, reduced stress, and electrostatic repulsion between interactive atoms, and rapid ion diffusion in insertion‐type electrode materials.^[^ ^42^, ^73^ ^]^ Oxygen vacancy, as a type of intrinsic defect, can be infused to modify the crystalline structure and electronic properties of a Wadsley–Roth phase anodic material and substantially improve the Li^+^ storage performance.

Among the many methods to introduce oxygen defects, high‐temperature treatment in a reductive or inert atmosphere is the most commonly used method.^[^ ^42^, ^73^ ^]^ Deng et al. proposed the assembly of Ti_2_Nb_10_O_29_ on highly conductive bacterial cellulose carbon (BCC). Oxygen vacancies are generated in the final BCC/Ti_2_Nb_10_O_29−_ *_x_* after annealing the compound at 500 °C for 1 h in a H_2_ atmosphere.^[^ ^74^ ^]^ The H_2_ reduces Ti^4+^ and Nb^5+^ to lower valence states and adjusts the surface chemical nature, this has been confirmed using X‐ray photoelectron spectroscopy, Electron paramagnetic resonance, and XANES spectra. DFT calculations show that the band gap of O_2_‐vacancy‐modulated Ti_2_Nb_10_O_29−_ *_x_* is 1.0 eV, which is lower than that of pristine Ti_2_Nb_10_O_29_ (≈1.81 eV). This demonstrates the improvement of electronic conductivity by the enhancement of electron density. The resultant BCC/Ti_2_Nb_10_O_29−_ *_x_* electrode delivers a high capacity of 253 mAh g^−1^ at 10 C after 500 cycles. In another study, Deng et al. developed a dual‐functional, low‐temperature carbon‐coating method that decomposes acetylene (C_2_H_2_ = 2C + H_2_) to simultaneously produce an oxygen vacancy in Ti_2_Nb_10_O_29_.^[^ ^45^ ^]^ In addition to the enhanced electronic conductivity, the Li+ diffusion energy barrier of defective Ti_2_Nb_10_O_29−_ *_x_* (0.71 eV) is also lower than that of perfect Ti_2_Nb_10_O_29_ (0.91 eV), indicating the easier Li^+^ migration. The regulation of oxygen defects might even weaken the lattice dynamical anisotropy during the cycling and lead to better structural stability, as demonstrated by the in situ synchrotron high‐energy XRD.

The use of reductive chemical agents can also cause defects on the surface of metal oxides. Zhang et al. synthesized the oxygen vacancy‐rich TiNb_2_O_7_ using the supercritical ethanol treatment at 250 °C for 1 h.^[^ ^75^ ^]^ The relative content of oxygen vacancies introduced by this strategy is nearly twice of that prepared by Ar atmosphere annealing, leading to the enhanced pseudo‐capacitance effect. Accordingly, the as‐prepared TiNb_2_O_7−_ *_x_* exhibits a high initial discharge capacity of 231 mAh g^−1^ at 0.1 A g^−1^ due to the optimized active sites.

Although many previous studies have demonstrated that oxygen vacancies can improve the electrochemical performance of Wadsley–Roth phase anode materials, there is also disagreement in the literature. A very recent perspective was put forth by Griffith et al. showing that the oxygen defect theory has more counter‐evidence than supporting‐evidence and may be oversimplified.^[^ ^76^ ^]^ The existence of extended planar intergrowth defects (Wadsley defects) has been proved to be reasonable in Wadsley–Roth phase materials.^[^ ^77^ ^]^ However, point defects such as oxygen vacancies in Wadsley–Roth crystallographic shear structures are largely absent due to the oxygen‐vacancy‐compensating nature of Wadsley defects.^[^ ^78^ ^]^ Therefore, the practical function of the oxygen vacancy modification strategy should be further examined carefully.

### 4.5 Challenges of Developing Large‐Scale Synthesis Methods

Through the above summarized modification strategies of structural engineering, carbon modification, ion doping, and oxygen vacancies, the electrochemical performance of Wadsley–Roth phase anode materials has been significantly enhanced. However, developing large‐scale synthesis methods also should be carefully considered for realizing their practical application in LIBs. As shown in **Table** **2**, various methods have been used to synthesize Wadsley–Roth phase anode materials. The most commonly used hydrothermal reaction and electrospinning methods can produce nanosized particles with variety of microstructures, but the complicated production process and relatively low production rate limit their wide application. Solid‐state reaction and solution precipitation methods are facile and easy‐controlled, which have been generally adopted to prepare most electrode materials in modern LIB industries. Nonetheless, there is still a gap between the rate capability of Wadsley–Roth phase anode materials prepared by these two methods and those synthesized by the hydrothermal reaction and electrospinning methods. Therefore, investigating the key factors such as starting material, sintering condition, precipitation condition, etc., to improve the crystallinity and dispersibility during the solid‐state reaction and solution precipitation processes is of great value, aiming to further improving the high rate performance of Wadsley–Roth phase anode materials. Besides, the new‐emerging synthesis methods of spray‐drying and solution combustion have been also developed, which show considerable potentials in combining the advantages of morphology control and simple production.

**Table 2:** The summarization of preparation methods of Wadsley–Roth phase anode materials and their capacities at low and high rates in recently published work

Synthesis methods	Materials	Voltage range [V vs Li^+^/Li]	Reversible specific capacity	High‐rate capability
Solid‐state reaction	N‐C@MSC‐Nb_2_O_5_^[^ ^32^ ^]^	1.0–3.0	250 mAh g^−1^ at 4 A g^−1^	≈177 mAh g^−1^ at 4 A g^−1^, 120 mAh g^−1^ at 16 A g^−1^
	Cr^3+^ and Nb^5+^ co‐doped Ti_2_Nb_10_O_29_^[^ ^70a^ ^]^	0.8–3.0	322 mAh g^−1^ at 0.1 C	206 mAh g^−1^ at 10 C
	Cr_0.2_Fe_0.8_Nb_11_O_29_^[^ ^71^ ^]^	0.8–3.0	254 mAh g^−1^ at 0.1 C	123 mAh g^−1^ at 10 C
	Fe_0.8_Mn_0.2_Nb_11_O_29_^[^ ^72a^ ^]^	0.8–3.0	≈288 mAh g^−1^ at 0.5 C	≈128 mAh g^−1^ at 10 C
	Fe_0.8_V_0.2_Nb11O_29_^[^ ^72a^ ^]^	0.8–3.0	≈275 mAh g^−1^ at 0.5 C	≈155 mAh g^−1^ at 10 C
	Cu_0.02_Ti_0.94_Nb_2.04_O_7_^[^ ^72b^ ^]^	0.8–3.0	315 mAh g^−1^ at 0.1 C	182 mAh g^−1^ at 10 C
	TiNb_24_O_62_^[^ ^9^ ^]^	1.0–3.0	≈207 mAh g^−1^ at 0.2 C	≈116 mAh g^−1^ at 10 C, ≈104 mAh g^−1^ at 15 C
	Nb_16_W_5_O_55_^[^ ^10^ ^]^	1.0–3.0	225 mAh g^−1^ at 0.2 C	≈166 mAh g^−1^ at 10 C, ≈50 mAh g^−1^ at 60 C
	TiNb_2_O_7_^[^ ^26^ ^]^	1.0–3.0	≈340 mAh g^−1^ at 0.2 C	≈175 mAh g^−1^ at 10 C, ≈61 mAh g^−1^ at 100 C
	FeNb_11_O_29_^[^ ^42b^ ^]^	0.8–3.0	≈400 mAh g^−1^ at 0.5 C	≈200 mAh g^−1^ at 10 C
	PNb_9_O_25_^[^ ^52a^ ^]^	1.0–3.0	≈235 mAh g^−1^ at 0.1 C	≈151 mAh g^−1^ at 10 C, ≈30 mAh g^−1^ at 60 C,
Sol–gel method	TiNb_2_O_7_ nanowires^[^ ^59^ ^]^	1.0–3.0	230.3 mAh g^−1^ at 0.1 A g^−1^	181.7 mAh g^−1^ at 3 A g^−1^, 168.1 mAh g^−1^ at 6 A g^−1^
Solution precipitation method	Nano‐TiNb_2_O_7_/CNTs composite^[^ ^66^ ^]^	0.8–3.0	346 mAh g^−1^ at 0.1 C	223 mAh g^−1^ at 10 C, 163 mAh g^−1^ at 30 C
	TiCr_0.5_Nb_10.5_O_29_/CNTs nanocomposite^[^ ^70b^ ^]^	0.8–3.0	297 mAh g^−1^ at 0.1 C	≈233 mAh g^−1^ at 10 C, 206 mAh g^−1^ at 0.1 C
Spray‐drying method	Nanoporous TiNb_2_O_7_/C composite microspheres^[^ ^64^ ^]^	0–3.0	350 mAh g^−1^ at 0.25 C	230 mAh g^−1^ at 10 C, 120 mAh g^−1^ at 30 C
Solution combustion method	Micro‐size Nb_14_W_3_O_44_^[^ ^27^ ^]^	1.0–3.0	221.3 mAh g^−1^ at 0.5 C	134.7 mAh g^−1^ at 10 C, 57.7 mAh g^−1^ at 100 C
Biomass template method	2D‐Ti_2_Nb_10_O_29_^[^ ^63^ ^]^	1.0–2.5	264 mAh g^−1^ at 0.5 C	≈220 mAh g^−1^ at 10 C, 171 mAh g^−1^ at 10 C
	3D/2D cross‐linked Ti_2_Nb_10_O_29−_ *_x_*@C composites^[^ ^45^ ^]^	1.0–2.5	≈300 mAh g^−1^ at 1 C	226 mAh g^−1^ at 10 C, 197 mAh g^−1^ at 20 C
Hydrothermal reaction	Hollow TiNb_2_O_7_@C spheres^[^ ^60b^ ^]^	0–3.0	289.4 mAh g^−1^ at 0.25 C	157.9 mAh g^−1^ at 10 C
	Mesoporous Ti_2_Nb_10_O_29_ microspheres^[^ ^61a^ ^]^	1.0–2.5	273.3 mAh g^−1^ at 10 C	212.8 mAh g^−1^ at 10 C, 171.4 mAh g^−1^ at 10 C
	Porous Ti_2_Nb_10_O_29_ nanospheres^[^ ^61b^ ^]^	0.8–2.5	312 mAh g^−1^ at 0.1 C	240 mAh g^−1^ at 10 C, 208 mAh g^−1^ at 20 C
	H‐Nb_2_O_5_ microspheres^[^ ^61c^ ^]^	1.0–3.0	164.1 mAh g^−1^ at 1 C	85.2 mAh g^−1^ at 10 C
	Nanosheet‐based Nb_12_O_29_ microspheres^[^ ^61d^ ^]^	0.8–3.0	282 mAh g^−1^ at 0.5 C	210 mAh g^−1^ at 10 C
	Mesoporous TiNb_2_O_7_ microspheres^[^ ^61e^ ^]^	1.0–3.0	258 mAh g^−1^ at 10 C	179 mAh g^−1^ at 10 C, 91 mAh g^−1^ at 30 C
	TiO_2_/Nb_2_O_5_/TiNb_2_O_7_ microspheres^[^ ^61f^ ^]^	1.0–2.5	255.3 mAh g^−1^ at 0.3 C	192.6 mAh g^−1^ at 5 C
	Carbon‐coated Nb_2_O_5_/TiNb_2_O_7_ porous spheres^[^ ^61g^ ^]^	1.0–3.0	282 mAh g^−1^ at 0.1 C	186 mAh g^−1^ at 10 C
	Porous TiNb_2_O_7_ nanospheres^[^ ^62^ ^]^	1.0–3.0	237.7 mAh g^−1^ at 1 C	179.1 mAh g^−1^ at 10 C, 94.9 mAh g^−1^ at 10 C
	Vertical graphene/Ti_2_Nb_10_O_29_/hydrogen molybdenum bronze composite arrays^[^ ^65^ ^]^	1.0–2.5	317 mAh g^−1^ at 2 C	267 mAh g^−1^ at 10 C, 163 mAh g^−1^ at 60 C
	Vertical graphene /TiNb_2_O_7_@S–C arrays^[^ ^68^ ^]^	1.0–2.5	284 mAh g^−1^ at 1 C	248 mAh g^−1^ at 10 C, 181 mAh g^−1^ at 160 C
	Carbon nanofiber/Ti_2_Nb_10_O_29_ core–shell arrays^[^ ^69^ ^]^	1.0–2.5	308 mAh g^−1^ at 1 C	241 mAh g^−1^ at 10 C, 188 mAh g^−1^ at 60 C
	Cr‐doped Ti_2_Nb_10_O_29_@vertical graphene@TiC‐C arrays^[^ ^70c^ ^]^	1.0–2.5	≈302 mAh g^−1^ at 1 C	260 mAh g^−1^ at 10 C, 220 mAh g^−1^ at 40 C
	N‐doped Ti_2_Nb_10_O_29_ with oxygen defect^[^ ^42e^ ^]^	1.0–2.5	320 mAh g^−1^ at 1 C	≈263 mAh g^−1^ at 10 C, 165 mAh g^−1^ at 100 C
	Ti_2_Nb_10_O_29−_ *_x_* mesoporous microspheres^[^ ^73a^ ^]^	0.8–3.0	309 mAh g^−1^ at 0.1 C	≈275 mAh g^−1^ at 10 C, 235 mAh g^−1^ at 40 C
	Bacterial cellulose carbon/Ti_2_Nb_10_O_29−_ *_x_* ^[^ ^74^ ^]^	1.0–2.5	≈300 mAh g^−1^ at 2 C	260 mAh g^−1^ at 10 C, 160.5 mAh g^−1^ at 40 C
	TiNb_2_O_7_ with oxygen defect^[^ ^75^ ^]^	1.0–3.0	205 mAh g^−1^ at 0.1 A g^−1^	≈165 mAh g^−1^ at 0.8 A g^−1^, 145.7 mAh g^−1^ at 1.6 A g^−1^
	TiNb_2_O_7_ nanoparticles^[^ ^38^ ^]^	0.6–3.0	≈322 mAh g^−1^ at 0.06 A g^−1^	215 mAh g^−1^ at 4.5 A g^−1^, 201 mAh g^−1^ at 6 A g^−1^
	CrNb_11_O_29_ nanorods^[^ ^42c^ ^]^	0.8–3.0	337 mAh g^−1^ at 0.1 C	228 mAh g^−1^ at 10 C
	Porous MoNb_12_O_33_ microspheres^[^ ^80^ ^]^	0.8–3.0	321 mAh g^−1^ at 0.1 C	200 mAh g^−1^ at 10 C
	N‐doped carbon‐Ti_2_Nb_10_O_29_@TiC/C core/shell arrays^[^ ^81^ ^]^	1.0–2.5	318 mAh g^−1^ at 1 C	≈257 mAh g^−1^ at 10 C, 202 mAh g^−1^ at 50 C
Electrospinning method	Nb_14_W_3_O_44_ nanowires^[^ ^57^ ^]^	1.0–3.0	228.3 mAh g^−1^ at 0.1 A g^−1^	137.8 mAh g^−1^ at 0.7 A g^−1^
	AlNb_11_O_29_ nanowires^[^ ^42a^ ^]^	0.8–3.0	256 mAh g^−1^ at 0.5 C	192 mAh g^−1^ at 10 C
	TiNb_24_O_62_ nanowires^[^ ^50^ ^]^	1.0–3.0	218 mAh g^−1^ at 0.5 C	189.6 mAh g^−1^ at 3 C, 177 mAh g^−1^ at 6 C
	Nb_12_WO_33_ nanowires^[^ ^48^ ^]^	1.0–3.0	220.8 mAh g^−1^ at 0.1 A g^−1^	145.8 mAh g^−1^ at 0.7 A g^−1^
	Interconnected Ti_2_Nb_10_O_29_ nanoparticles^[^ ^58^ ^]^	1.0–3.0	290 mAh g^−1^ at 0.2 A g^−1^	138 mAh g^−1^ at 10 A g^−1^, 93 mAh g^−1^ at 15 A g^−1^
	TiNb_2_O_7_ hollow nanofibers^[^ ^36b^ ^]^	1.0–3.0	291.7 mAh g^−1^ at 0.4 C	196.5 mAh g^−1^ at 10 C
	FeNb_11_O_29_ nanotubes^[^ ^44^ ^]^	1.0–3.0	233.2 mAh g^−1^ at 1 C	142.3 mAh g^−1^ at 10 C, 53.4 mAh g^−1^ at 50 C
	TiNb_2_O_7_ nanotubes^[^ ^60a^ ^]^	1.0–3.0	≈266 mAh g^−1^ at 1 C	≈224 mAh g^−1^ at 10 C, ≈180 mAh g^−1^ at 100 C
	Ti_2_Nb_10_O_29_ hollow nannofibers^[^ ^60c^ ^]^	0.8–3.0	307 mAh g^−1^ at 0.1 C	176 mAh g^−1^ at 10 C, 136 mAh g^−1^ at 10 C
	CrNb_49_O_124_ nanotubes^[^ ^60d^ ^]^	1.0–3.0	340 mAh g^−1^ at 0.06 A g^−1^	≈190 mAh g^−1^ at 0.5 A g^−1^
	Hybrid Ti_2_Nb_10_O_29_ **/**carbon nanofiber mats^[^ ^67^ ^]^	0.8–3.0	≈257 mAh g^−1^ at 0.1 C	≈161 mAh g^−1^ at 10 C, ≈137 mAh g^−1^ at 20 C
	GaNb_11_O_29_ Nanowebs^[^ ^42d^ ^]^	0.8–3.0	264 mAh g^−1^ at 0.1 C	175 mAh g^−1^ at 10 C
	VNb_9_O_25_ nanowires^[^ ^53b^ ^]^	1.0–3.0	207.2 mAh g^−1^ at 0.1 A g^−1^	117.3 mAh g^−1^ at 0.5 A g^−1^
	ZrNb_14_O_37_ Nanowires^[^ ^82^ ^]^	1.0–3.0	236.4 mAh g^−1^ at 0.1 A g^−1^	190.9 mAh g^−1^ at 0.5 A g^−1^, 168.2 mAh g^−1^ at 0.7 A g^−1^
	Zn_2_Nb_34_O_87_ nanofibers^[^ ^83^ ^]^	0.8–3.0	310 mAh g^−1^ at 0.1 C	219 mAh g^−1^ at 10 C

From the perspective of scalable production, the cost and abundance of niobium should be also considered. The price of Nb_2_O_5_ (USD 156/100 g, 99.9% trace metals basis, Sigma‐Aldrich) is higher than TiO_2_ (USD 84.5/100 g, rutile, 99.9% trace metals basis, Sigma‐Aldrich). However, the synthesis of Li_4_Ti_5_O_12_ requires the addition of lithium compounds such as Li_2_CO_3_ (USD 60.2/100 g, ≥99.0%, Sigma‐Aldrich). Thus, the cost of raw materials for producing Li_4_Ti_5_O_4_ may be close to that for producing Nb‐based oxides. The development of LIB technology is always accompanied by trade‐offs; there is no known perfect electrode material. Considering that Wadsley–Roth phase anode materials possess higher volumetric energy densities than Li_4_TiO_5_, the slightly increased material cost is accepted. Because the volumetric energy density is a crucial indicator, which is even more important than the gravimetric energy density for most applications.

According to the U.S. Geological Survey, “the estimated global reserves and resources of niobium are large and appear more than sufficient to meet global demand for the foreseeable future, possibly the next 500 years.”^[^ ^79^ ^]^ Therefore, the reserves of niobium can entirely meet the demand for producing potential Wadsley–Roth phase Nb‐based oxides.

## 5 Implementation in Full Cells

To evaluate the feasibility and commercial perspective of Wadsley–Roth phase anode materials, the electrochemical performance of full cells with such anodes must be tested. As mentioned in the introduction, conventional graphite anodes suffer from poor rate capability and potential Li‐plating risks, and hence, cannot satisfy the increasing requirements for high‐output LIBs. Recently developed LIBs with Li_4_Ti_5_O_12_ anodes (such as the “SCiB” launched by Toshiba) have exhibited outstanding rate performance and improved safety properties.^[^ ^84^ ^]^ Wadsley–Roth phase anode materials not only have lithiation potentials similar to those of Li_4_Ti_5_O_12_, but also have a higher theoretical specific capacity. Therefore, exploring high‐power and high‐safety lithium‐ion full cell systems with Wadsley–Roth phase anodes is feasible.

The major commercial cathode materials for LIBs are LiCoO_2_, LiNi*_x_*Mn*_y_*Co*_z_*O_2_, LiFePO_4_, LiMn_2_O_4_, and LiNi_0.5_Mn_1.5_O_4_, which show varying electrochemical characteristics. In an ideal full cell design, the capacity of the positive electrode (*Q*_p_ = *C*_p_ (specific capacity of cathode material) × *m*_p_ (mass of cathode material)) must be matched with that of the negative electrode (*Q*_n_ = *C*_n_ (specific capacity of anode material) × *m*_n_ (mass of anode material)), and the capacity of the cell (*Q*_cell_) follows the law: 1/*Q*_cell_ = 1/*Q*_p_ + 1/*Q*_n_. The value of the average working voltage (*U_cell_*) can be further used to calculate the energy density of the cell (*E_cell_*), i.e., *E_cell_ = Q_cell_×U_cell_*. In practical LIBs, the N/P (*Q*_n_/*Q*_p_) ratio should be critically investigated to obtain a balance between cycling stability, safety feature, and cost consideration.^[^ ^85^ ^]^ In this section, we detail the electrochemical performance and matching principles of Wadsley–Roth phase anode materials‐based full cell assemblies using different cathodes.

### 5.1 Full Cells Coupled with 4 V Cathodes

#### 5.1.1 LiCoO_2_ and LiNi_1−_ *_x_*_−_ *_y_*Co*_y_*Mn*_x_*O_2_ as Cathodes

LiCoO_2_ is a first‐generation commercialized cathode material, and is still widely used in portable devices because of its high tap density (3.6–4.2 g cm^−3^). Its operating voltage is ≈3.9 V (vs Li^+^/Li), with an available capacity of ≈138 mAh g^−1^ (corresponding to the extraction of 0.5 Li^+^). Li et al. studied the electrochemical performance of a full cell constructed using ZrNb_14_O_37_ nanowires and commercial LiCoO_2_.^[^ ^82^ ^]^ The as‐fabricated ZrNb_14_O_37_//LiCoO_2_ cell had an average voltage output of ≈2.17 V, and a reversible capacity of 84 mAh g^−1^ (*C*_p_, based on the mass of cathode). The cell retains 81.3% of capacity after 100 charge/discharge cycles at 0.1 A g^−1^.

Layered Ni–Co–Mn oxides (LiNi_1−_ *_x_*_−_ *_y_*Co*_y_*Mn*_x_*O_2_) consist of closely packed oxygen atoms with alternating layers of transition metals and Li in the interstitial sites. The main advantages of LiNi_1−_ *_x_*_−_ *_y_*Co*_y_*Mn*_x_*O_2_ are its increased capacity, low toxicity, and reduced cost due to the replacement of expensive Co with inexpensive Ni and Mn. The available capacity of LiNi_1−_ *_x_*_−_ *_y_*Co*_y_*Mn*_x_*O_2_ varies from 155 to 220 mAh g^−1^ according to the composition. Takami and co‐workers, of the Toshiba Corporation, fabricated large‐size TiNb_2_O_7_@C//LiNi_0.6_Co_0.2_Mn_0.2_O_2_ batteries with a capacity of 49 Ah.^[^ ^86^ ^]^ The cell exhibited a high volumetric energy density of 350 Wh L^−1^, ultralong cycle life (capacity retention after 7000 cycles at 1 C is as high as 86%), and excellent fast‐charging characteristics (the time to reach 90% SoC is less than 6 min at 10 C), which should make a viable prototype for commercial EV applications.

#### 5.1.2 LiMn_2_O_4_ as the Cathode

The low‐cost and eco‐friendly LiMn_2_O_4_ with a 3D spinel structure was first reported as an alternative to LiCoO_2_.^[^ ^87^ ^]^ The discharging voltage of LiMn_2_O_4_ (≈4.0 V vs Li^+^/Li) is slightly higher than that of LiCoO_2_, but its available capacity is much lower (≈110 mAh g^−1^). Zhu et al. synthesized porous Nb_12_MoO_33_ microspheres with diameters in the range of 1.5–3.0 µm and assembled as an anode into a full battery with a commercial LiMn_2_O_4_ cathode.^[^ ^80^ ^]^ The Nb_12_MoO_33_//LiMn_2_O_4_ cell exhibits a reversible capacity (*C*_n_, based on the mass of anode) of 234 mAh g^−1^ and a relatively high working voltage of ≈2.58 V at 0.1 C. In addition, a high capacity retention of 93.9% was achieved for the cell up to 1000 cycles at 5 C. Interestingly, Lakhnot et al. proposed a new type of aqueous LIB with niobium tungsten oxides as the anode, LiMn_2_O_4_ as the cathode, and 21 m LiTFSI (lithium bis(trifluoromethane sulfonyl)imide) aqueous solution as the water‐in‐salt electrolyte.^[^ ^88^ ^]^ Both the Nb_16_W_5_O_55_//LiMn_2_O_4_ and Nb_18_W_8_O_93_//LiMn_2_O_4_ cells can be cycled stably in the voltage window of 1.0–2.5 V. After 100 cycles at 1 C, the gravimetric capacity of Nb_18_W_8_O_93_ (*C*_n_) in the aqueous lithium‐ion full battery remains at 69 mAh g^−1^, showing a capacity retention of 88%, which is better than that of Nb_16_W_5_O_55_ (48 mAh g^−1^ with a capacity retention of 85%). Notably, the volumetric energy density of the Nb_18_W_8_O_93_ electrode can reach as high as 200 Ah L^−1^ due to the high tap density (2.21–2.97 g mL^−1^), which is superior to that of the majority anode materials in aqueous systems.

### 5.2 Full Cells Coupled with 3.5 V Cathodes

LiFePO_4_ is a cathode material that crystallizes into an olivine structure. Its theoretical capacity based on one‐electron transfer is 170 mAh g^−1^, with a stable potential plateau of 3.5 V (vs Li^+^/Li).^[^ ^89^ ^]^ The differential scanning calorimetry measurements of overcharged layered, spinel, and olivine cathodes demonstrate the superior thermal stability of LiFePO_4_.^[^ ^90^ ^]^ Therefore, matching the LiFePO_4_ cathode with Wadsley–Roth phase anodes may prevent the risk of thermal runaway by a substantial margin. Deng et al. demonstrated a full cell based on the VG/Ti_2_Nb_10_O_29_/HMB (vertical graphene/Ti_2_Nb_10_O_29_/hydrogen molybdenum bronze) core/shell sandwich arrays anode and a LiFePO_4_ cathode.^[^ ^65^ ^]^ The full cell exhibited a high discharge capacity (*C*_n_) of 314 mAh g^−1^ at 0.5 C, and about 82% capacity retention after 200 cycles at 5 C.

Yao et al. studied the Li^+^ storage performance of NC‐TiNb_12_O_29_@TiC‐C anodes combined with LiFePO_4_ cathodes (TiNb_12_O_29_ nanoparticles are sandwiched between an N‐doped C shell and a TiC/C nanowire backbone).^[^ ^81^ ^]^ The operating voltage of the TiNb_12_O_29_@TiC‐C//LiFePO_4_ cell is close to about 1.7 V. It retains a capacity of 125 mAh g^−1^ (*C*_n_) at a high current density of 10 C after 5000 cycles. Based on the total mass of the anode and the cathode, the cell delivers energy densities of 177 and 85 Wh kg^−1^ at power densities of 195 and 2000 W kg^−1^, respectively. The same group of authors also reported the use of Ti_2_Nb_10_O_29_@TiC/C‐NC (N‐doped Ti_2_Nb_10_O_29_ is loaded into a TiC/C‐NC core‐branch skeleton) as the anode.^[^ ^30^ ^]^ In addition to the Ti_2_Nb_10_O_29_‐based anodes, TiNb_2_O_7_ anodes compatible with the LiFePO_4_ cathode have also been investigated.^[^ ^42e^ ^]^ Apart from the aforementioned Ti_2_Nb_10_O_29_‐based anodes, TiNb_2_O_7_ anodes have also been investigated to match with LiFePO_4_ cathode.^[^ ^68^ ^]^ The as‐fabricated VG/TiNb_2_O_7_@S‐C//LiFePO_4_ full battery with an operating voltage of ≈1.65 V can maintain a capacity (*C*_n_) of 230 mAh g^−1^ after 1000 cycles at 10 C, corresponding to a capacity retention of 95.5%. In a previous study, we proposed a Nb_14_W_3_O_44_//LiFePO_4_ full cell configuration, and studied its electrochemical performance at an evaluated temperature of 50 °C.^[^ ^27^ ^]^ Under such an extreme condition, the discharge capacity of the Nb_14_W_3_O_44_//LiFePO_4_ full cell retained 100.5 mAh g^−1^ after 1000 cycles at 10 C. A high energy density of 143.8 Wh kg^−1^ is achieved in this cell configuration.

### 5.3 Full Cells Coupled with 5 V Cathodes

LiNi_0.5_Mn_1.5_O_4_ is a promising high‐voltage cathode material with a flat plateau of ≈4.7 V (vs Li^+^/Li) that can deliver a large capacity of 147 mAh g^−1^ based on the two‐electron reaction of Ni^2+^/Ni^4+^ redox couples, in which the residual Mn^4+^ ions are left electrochemically inert.^[^ ^91^ ^]^ Benefiting from the high discharge potential, the relatively high working voltage and power density are predictable while matching with Wadsley–Roth phase anode materials to construct full cells. Lin et al. fabricated an AlNb_11_O_29_//LiNi_0.5_Mn_1.5_O_4_ full cell that exhibited a high operating voltage of ≈3.05 V and a reversible capacity (*C*_n_) of 136 mAh g^−1^, with a high capacity retention of 93.2% after 100 cycles at 1 C.^[^ ^42a^ ^]^ They also comprehensively evaluated the electrochemical performance of Zn_2_Nb_34_O_87_ nanofibers//LiNi_0.5_Mn_1.5_O_4_ full cell.^[^ ^83^ ^]^ It exhibited a large capacity (*C*_n_) of 166 mAh g^−1^ at 1 C, and retained 92.3% of the initial capacity after 100 cycles. Even better cycling stability with only 0.0035% decay per cycle was obtained at 5 C for up to 1000 cycles. Han and Goodenough reported a TiNb_2_O_7_ anode combined with a LiNi_0.5_Mn_1.5_O_4_ cathode to assemble a 3‐V class TNO//LiNi_0.5_Mn_1.5_O_4_ full cell and studied the capacity matchup principles.^[^ ^92^ ^]^ The results indicated that the cycling stability of the anode‐limited battery is obviously better than that of the cathode‐limited one. Liang et al. fabricated a 18 650‐type full cell with a capacity of 1.2 Ah, by using a Mg‐doped LiNi_0.5_Mn_1.5_O_4_ cathode coupled with a TiNb_2_O_7_ anode.^[^ ^93^ ^]^ A flat operating voltage at ≈3.0 V and a minor polarization effect were observed in the TiNb_2_O_7_//Mg_0.1_‐LiNi_0.5_Mn_1.5_O_4_ cell. A capacity retention of ≈96.3% was achieved after 68 cycles at 0.1 C, which corresponds to a gravimetric energy density (*E*_cell_) of 91.4 Wh kg^−1^.

**Table** **3** summarizes a few electrochemical properties of the lithium‐ion full cells using anode materials discussed above. Some valuable information can be concluded after carefully examining these samples. 1) The negative‐limited full cells obviously deliver better cycling stability than those of the positive‐limited full cells. Because the voltage profile of the Wadsley–Roth phase anode is not flat, the cathode is easy to be overcharged due to the slight electrochemical polarization of Wadsley–Roth phase anode during cycling when the full cell is assembled by the positive‐limited principle. The overcharge process of LIB cathode materials results in the excessive extraction of Li^+^ ions, which will reduce the crystal stability of the host structures.^[^ ^94^ ^]^ 2) The TiNb_2_O_7_//LiNi_0.6_Co_0.2_Mn_0.2_O_2_ full cell shows the longest lifespan among all these battery systems.^[^ ^87^ ^]^ 3) The TiNb_2_O_7_//LiNi_0.5_Mn_1.5_O_4_ full cell exhibits the highest operating voltage and energy density.^[^ ^92^ ^]^ From the perspective of energy utilization efficiency, LiNi_0.5_Mn_1.5_O_4_ may be a more appropriate cathode to match with Wadsley–Roth phase anodes. However, the conventional carbonate‐based electrolyte is easy to decompose and many adverse side reactions may occur between the cathode/electrolyte interface due to the high working voltage of LiNi_0.5_Mn_1.5_O_4_, which will exert negative influence on the cycling stability and safety performance.^[^ ^95^ ^]^

**Table 3:** Summary of recently published studies on the electrochemical performance of Wadsley–Roth phase anode‐based full cell LIBs (*C*_p_ represents the positive‐limited capacity, *C*_n_ represents the negative‐limited capacity, and *E*_cell_ represents the energy density calculated based on the total mass of active materials in both anode and cathode)

Anode	Cathode	Mass ratio (anode:cathode)	N/P ratio	Operating voltage [V]	Specific capacity	Energy density (*E*_cell_)	Cycling stability
ZrNb_14_O_37_^[^ ^82^ ^]^	LiCoO_2_	1:1.8	N/A	2.17	*C*_p_ = 99.6 mAh g^−1^ at 0.1 A g^−1^	138.9 Wh kg^−1^ at 0.1 C	81.3% after 100 cycles at 0.1 A g^−1^,
TiNb_2_O_7_@C^[^ ^86^ ^]^	LiNi_0.6_Co_0.2_Mn_0.2_O_2_	N/A	N/A	2.2	N/A	350 Wh L^−1^ at 1 C	86% after 7000 cycles at 10 C
Nb_12_MoO_33_^[^ ^80^ ^]^	LiMn_2_O_4_	1:3	N/A	≈2.58	*C*_n_ = 234 mAh g^−1^ at 0.1 C	150.9 Wh kg^−1^ at 0.1 C	93.9% after 1000 cycles at 5 C
VG/Ti_2_Nb_10_O_29_/HMB^[^ ^65^ ^]^	LiFePO_4_	2:13	N/A	≈1.7	*C*_n_ = 314 mAh g^−1^ at 0.5 C	≈71.2 Wh kg^−1^ at 0.5 C	82% after 200 cycles at 5 C
NC‐TiNb_12_O_29_@ TiC‐C^[^ ^81^ ^]^	LiFePO_4_	N/A	1:1.15	≈1.7	*C*_n_ = 255 mAh g^−1^ at 1 C	≈146.7 Wh kg^−1^ at 1 C	88.6% after 5000 cycles at 10 C
Ti_2_Nb_10_O_29_@TiC/C‐NC^[^ ^42e^ ^]^	LiFePO_4_	1:2.7	N/A	≈1.7	*C*_n_ = 269 mAh g^−1^ at 1 C	≈123.6 Wh kg^−1^ at 1 C	≈52% after 2000 cycles at 20 C
VG/TiNb_2_O_7_@S‐C^[^ ^68^ ^]^	LiFePO_4_	N/A	1:1.1	≈1.65	*C*_n_ = 280 mAh g^−1^ at 1 C	≈151.3 Wh kg^−1^ at 1 C	95.5% after 1000 cycles at 10 C
Nb_14_W_3_O_44_^[^ ^27^ ^]^	LiFePO_4_	N/A	0.8–0.9:1	≈1.8	*C*_n_ = 226.9 mAh g^−1^ at 0.5 C	143.8 Wh kg^−1^ at 0.5 C	≈91.1% after 1000 cycles at 10 C
TiNb_24_O_26_^[^ ^50^ ^]^	LiFePO_4_	2.27:1	N/A	≈1.65	*C*_p_ = 112.7 mAh g^−1^ at 1 C	56.9 Wh kg^−1^ at 0.5 C	≈95.5% after 50 cycles at 1 C
FeNb_11_O_29_^[^ ^44^ ^]^	LiFePO_4_	N/A	N/A	≈1.7	*C*_p_ = 119.9 mAh g^−1^ at 1 C	N/A	74.3% after 100 cycles at 1 C
AlNb_11_O_29_^[^ ^42a^ ^]^	LiNi_0.5_Mn_1.5_O_4_	1:2.8	N/A	≈3.05	*C*_n_ = 195 mAh g^−1^ at 0.1 C	156.5 Wh kg^−1^ at 0.1 C	93.2% after 100 cycles at 1 C
Zn_2_Nb_34_O_87_^[^ ^83^ ^]^	LiNi_0.5_Mn_1.5_O_4_	1:3	N/A	≈3.0	*C*_n_ = 236 mAh g^−1^ at 0.1 C	177 Wh kg^−1^ at 0.1 C	96.5% after 1000 cycles at 5 C
TiNb_2_O_7_^[^ ^92^ ^]^	LiNi_0.5_Mn_1.5_O_4_	N/A	1/1.2	≈3.0	*C*_n_ = 210.8 mAh g^−1^ at 0.1 C	≈220.1 Wh kg^−1^ at 0.1 C	95% after 50 cycles at 0.1 C
TiNb_2_O_7_^[^ ^93^ ^]^	Mg_0.1_‐LiNi_0.5_Mn_1.5_O_4_	13:25	N/A	≈3.0	N/A	94.87 Wh kg^−1^ at 0.1 C	96.3% after 68 cycles at 0.1 C

## 6 Conclusions and Outlook

The unique features of Wadsley–Roth phase niobium‐based oxides, including 3D open crystalline structure, moderate lithiation potential, and reversible redox couples, can usher in a new era of LIBs with high power density, long lifespan, and high safety. Despite this, there is still extensive advancement space for further enhancing their electrochemical kinetics. In this paper, we systematically analyzed structural features and electrochemical reaction mechanisms of various representative Wadsley–Roth phase anode materials and then explored the improvement strategies. We also summarized the electrochemical properties of a series of full cells that demonstrate the compatibility of Wadsley–Roth phase anode materials with commercial cathode materials to fabricate LIBs applicable for commercial use.

Yet, several significant aspects, such as Li^+^ storage mechanism, modification of electrode materials, full‐cell‐assembly parameters, safety considerations, and industrial feasibility should be considered in future studies.

Listed below are the recommendations following the findings of this study:

i) Researchers must comprehensively study the lithiation mechanism and electrode–electrolyte interface behavior of Wadsley–Roth phase anode materials using various in situ or operando techniques and theoretical methods. The relationship between the electrochemical performance, intrinsic structure, and physicochemical properties of the anode materials should be established to obtain a guideline for exploiting more precise and valid electrode‐modification strategies.
ii) For commercial applications, the areal specific capacity of anodes must be no less than 3.0 mAh cm^−2^, which means that the loading mass of Wadsley–Roth phase anode materials in the electrode should be more than 10 mg cm^−2^. Most of the existing material modification strategies for improving the rate performance and cycling stability are based on results obtained at much lower mass loading levels (≈1–2 mg cm^−2^). Whether or not these modification methods are effective at higher loading mass conditions remains unanswered. Besides, the initial Coulombic efficiency is also an important indicator for commercial LIBs, and it should be more than 90% for full cells to realize high energy density. However, this modification approach often causes serious side reactions at the interface, resulting in much lower initial Coulombic efficiency. Therefore, Wadsley–Roth phase anode materials must be optimized to achieve high power density and balance the gravimetric/volumetric energy densities at a high loading mass condition.
iii) The reduction of the electrolyte by the anode and thermal decomposition of the anode at elevated temperatures are the possible reactions that trigger the thermal runaway of batteries. It is plausible that the liberation heat related to the Wadsley–Roth phase anode materials may be apparently suppressed owing to their relatively high lithiation potentials, but the detailed thermal reaction mechanisms between Wadsley–Roth phase anode materials and electrolytes still need to be critically evaluated.
iv) There are still no criteria for assembling and testing Wadsley–Roth phase anodes‐based LIB full cells. Parameters, such as cell design (thickness and porosity of electrodes), electrode capacity balance (anode‐limited or cathode‐limited), battery capacity, and abuse tolerance (high‐ or low‐temperature cycling stability), lack any organized criteria. Moreover, the reliable safety features of Wadsley–Roth phase anode‐based LIB full cells must be systematically evaluated using characterizations such as accelerating rate calorimetry, nail penetration test, and overcharge test.
v) The use of high‐safety Wadsley–Roth phase anode materials in solid‐state electrolytes may be the ultimate route to address the safety issues pertaining to the carbonate‐solvent‐based LIBs. In addition, these anode materials can also be applied in well‐designed, noninflammable, aqueous electrolytes due to the moderate lithiation potentials that prevent the reduction of H^+^.

## Conflict of Interest

The authors declare no conflict of interest.

## Supporting information

Supporting Information
