# Supplementary material for: Wadsley–Roth Crystallographic Shear Structure Niobium‐Based Oxides: Promising Anode Materials for High‐Safety Lithium‐Ion Batteries
Source: Adv Sci (Weinh). 2021 Mar 15;8(12):2004855. doi: 10.1002/advs.202004855 (PMC8224428; doi:10.1002/advs.202004855)
Supplement: Supplementary file 1 — Supporting Information [file ADVS-8-2004855-s001.pdf]

© 2021 Wiley-VCH GmbH

ADVANCED SCIENCE
Open Access

Supporting Information

for *Adv. Sci.*, DOI: 10.1002/advs.202004855

Wadsley-Roth crystallographic shear structure niobium-based oxides: promising anode materials for high-safety lithium-ion batteries

*Yang Yang, Jinbao Zhao**

Supporting Information

Wadsley-Roth crystallographic shear structure niobium-based oxides: promising anode materials for high-safety lithium-ion batteries

Yang Yang,[1] Jinbao Zhao[2,]*

[1]School of Chemical Engineering and Light Industry, Guangdong University of Technology, Guangzhou 510006, P. R. China

[2]State Key Lab of Physical Chemistry of Solid Surfaces, State-Province Joint Engineering Laboratory of Power Source Technology for New Energy Vehicle,College of Chemistry and Chemical Engineering, Xiamen University, Xiamen, 361005, P. R. China

AUTHOR INFORMATION

Corresponding Author

*E-mail: jbzhao@xmu.edu.cn

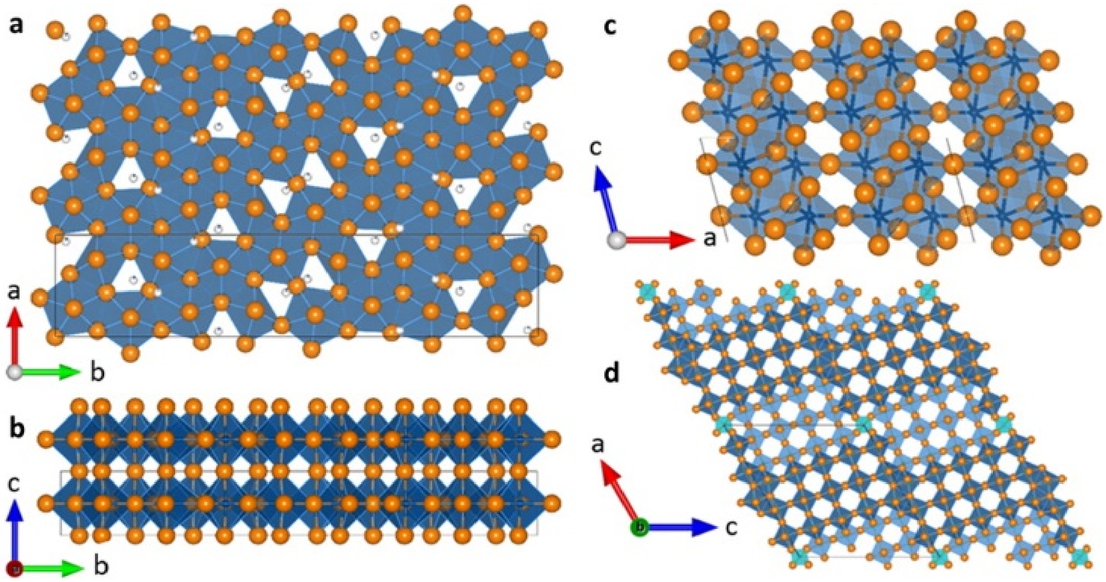

Figure S1. Crystal structures of (a,b) T-Nb_2O_5, (c) B-Nb_2O_5, and (d) H-Nb_2O_5.Reproduced with permission.[1]Copyright 2016, *American Chemical Society*.

References

[1] K. J. Griffith, A. C. Forse, J. M. Griffin, C. P. Grey, *J. Am. Chem. Soc.***2016**, 138, 8888.
